# Epigenetic remodeling via HDAC6 inhibition amplifies anti-tumoral immune responses in myeloid leukemia cells

**DOI:** 10.1038/s41419-026-08541-3

**Published:** 2026-03-07

**Authors:** Julian Schliehe-Diecks, Jia-Wey Tu, Pawel Stachura, Katerina Schaal, Marie Kemkes, Eleni Vasileiou, Nadine Rüchel, Danielle Brandes, Melina Vogt, Thomas Lenz, Adarsh Nair, Stefanie Scheu, Pilar M. Dominguez, Agata Pastorczak, Karin Nebral, Kai Stühler, Ute Fischer, Aleksandra A. Pandyra, Arndt Borkhardt, Sanil Bhatia

**Affiliations:** 1 Department of Pediatric Oncology, Hematology and Clinical Immunology, Medical Faculty, Heinrich Heine University Düsseldorf, Düsseldorf, Germany; 2 Institute of Clinical Chemistry and Clinical Pharmacology, University Hospital Bonn, Bonn, Germany; 3 German Center for Infection Research (DZIF), Partner Site Bonn-Cologne, Bonn, Germany; 4 Molecular Proteomics Laboratory, Biological Medical Research Center, Heinrich-Heine-University Düsseldorf, Düsseldorf, Germany; 5 Institute of Medical Microbiology and Hospital Hygiene, Medical Faculty and University Hospital Düsseldorf, Heinrich Heine University Düsseldorf, Düsseldorf, Germany; 6 Institute of Immunology, Rostock University Medical Center, Rostock, Germany; 7 Université Côte d’Azur, INSERM, C3M U1065, Nice, France; 8 Department of Genetic Predisposition to Cancer, Medical University of Lodz, Lodz, Poland; 9 St. Anna Children’s Cancer Research Institute (CCRI), Vienna, Austria; 10 Labdia Labordiagnostik, Vienna, Austria; 11 Institute for Molecular Medicine, Proteome Research, University Hospital and Medical Faculty, Heinrich-Heine-University Düsseldorf, Düsseldorf, Germany; 12 German Cancer Consortium (DKTK), Partner Site Essen/Düsseldorf, Düsseldorf, Germany

**Keywords:** Acute myeloid leukaemia, Chronic myeloid leukaemia

## Abstract

Histone deacetylase 6 (HDAC6) has emerged as a promising therapeutic target in cancer due to its immunomodulatory effects. While its prognostic significance remains debated, we demonstrate that HDAC6 loss significantly impairs myeloid leukemia progression in vivo, despite having no functional impact on leukemia cell proliferation in vitro. Global proteome and secretome profiling of HDAC6-knockout (KO) cells revealed upregulation of several immune-related modulators, including RNase T2, a tumor suppressor known to modulate the tumor microenvironment. Notably, RNase T2 upregulation upon HDAC6 loss was observed in myeloid leukemia cells but not in lymphoblastic leukemia cells. Moreover, pharmacological inhibition of HDAC6 recapitulated this phenotype, leading to RNase T2 upregulation in myeloid leukemia cells. ATAC-seq revealed increased chromatin accessibility of RNase T2 following HDAC6 loss, highlighting a functionally epigenetic regulatory contribution. Further functional assays conducted in an immunocompetent setting, both ex vivo and in vivo, demonstrated that HDAC6 inhibition sensitized murine myeloid leukemia cells to broad CD8^+^ T cell activation as evidenced by increased TNFα and CD107a expression. Consistently, in a syngeneic murine model, HDAC6 inhibition restricted the growth of myeloid leukemia cells. Moreover, an extended drug screening analysis identified Cytarabine and Clofarabine as significantly synergizing with HDAC6 inhibitor (Ricolinostat) in myeloid leukemia cell lines and in patient-derived xenograft (PDX) cells, while showing limited synergy in lymphoid leukemia cell lines, PDX, or healthy control cells. These findings suggest that HDAC6 represents a promising therapeutic target in myeloid lineage-derived leukemia cells by simultaneously enhancing immune activation and increasing chemosensitivity.

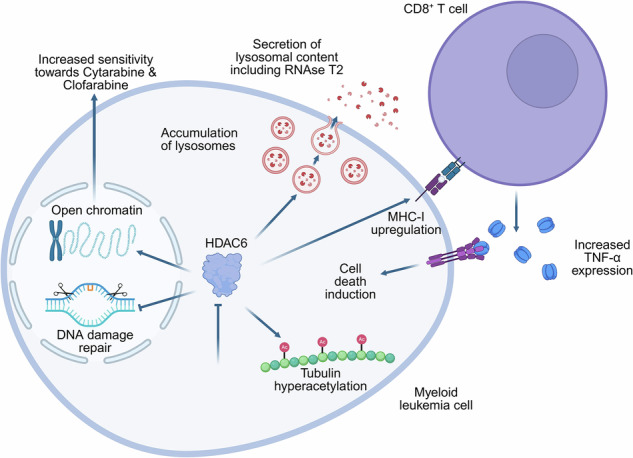

## Introduction

Aberrant epigenetic modifications, including histone deacetylation, play a key role in the leukemogenesis of acute myeloid leukemia (AML) and contribute to chemotherapy resistance by silencing tumor suppressor genes regulating chemosensitivity [1]. Dysregulation of HDACs has been linked to impaired hematopoietic differentiation, cell cycle defects, DNA damage accumulation, and reduced cell viability [2]. Although mutations in HDACs are uncommon in myeloid malignancies, dysregulated expression and aberrant recruitment by leukemia-associated fusion proteins have been reported [3, 4]. HDAC inhibitors (HDACi) have recently emerged as a promising class of therapeutics for various malignancies, including AML [5–7]. However, the approved HDACi for treating hematological malignancies are predominantly pan-HDACi, which broadly inhibit multiple HDAC isoforms involved in essential cellular functions [8, 9]. This lack of specificity leads to a range of adverse effects, including cardiac toxicity and severe gastrointestinal disturbances, some of which can be life-threatening [10–12]. These toxicities often require the discontinuation of HDACi-based therapies to prevent severe complications. Additionally, in clinical practice, HDACi are rarely used as monotherapy and are typically combined with other therapeutic agents, adding complexity to treatment regimens [13, 14]. Selective targeting of specific HDAC isoforms may offer a promising approach to restricting their activity to particular cellular processes, potentially reducing toxicity and enhancing therapeutic tolerability.

Among the various HDAC isoforms, HDAC6 has emerged as a particularly compelling therapeutic target due to its distinct biological roles and relevance in oncogenesis [15]. HDAC6 is primarily localized in the cytoplasm and regulates a range of cellular functions through substrate deacetylation [16–19]. However, HDAC6-deficient mice remain viable and develop normally, supporting its potential as a safe therapeutic target [20]. HDAC6 is found to be overexpressed across various hematologic malignancies [21–23]. Pharmacological HDAC6 inhibition has been shown to target leukemic stem cells (LSCs) in chronic myeloid leukemia (CML) [22, 24, 25]. A recent study from a zebrafish model demonstrates that inhibition of HDAC6 suppresses LSC expansion and reduces the proliferation of AML cell lines [26], underscoring its potential as a therapeutic target across myeloid malignancies [27]. However, the prognostic relevance of HDAC6 as a therapeutic target remains debated, as HDAC6 inhibition alone has been reported to exert minimal or no cytotoxic effects on leukemia cells in ex vivo studies [28–30].

Through a database-driven approach, we identified HDAC6 as a promising candidate in AML. Using genetic models and employing multi-omics characterization, we identified RNase T2, a known activator of pro-inflammatory signaling, to be upregulated upon targeting HDAC6. Further, our ex vivo and in vivo studies demonstrated that HDAC6 inhibition enhanced broad CD8^+^ T cell activation and, therefore, anti-leukemic responses. Beyond its immunomodulatory role, we found that HDAC6 inhibition sensitized myeloid leukemia cells to standard chemotherapeutics. These findings highlight the potential of HDAC6 as a target in myeloid leukemia, underscoring the need for further investigation into its clinical applicability.

## Results

### HDAC6 ablation impairs in vivo growth of (K562 and MV4-11) myeloid leukemia cells

We data-mined clinical data using TARGET [31] and TCGA-LAML [32] datasets, and identified prognostic implications. High HDAC6 expression significantly correlated with poorer survival in AML, whereas in acute lymphoblastic leukemia (ALL), elevated HDAC6 levels were associated with improved survival outcomes (Supplementary Fig. S1A–H). These findings contrast with earlier (ex vivo) studies suggesting that selective HDAC6 inhibition has minimal or no cytotoxic effects on the leukemia cells [28–30]. Further, in vitro CRISPR-based dependency analysis (using data from the DepMap portal) also suggests that HDAC6 exhibits the lowest dependency among HDAC family members in leukemia cells (Supplementary Fig. S1I).

**Fig. 1:**
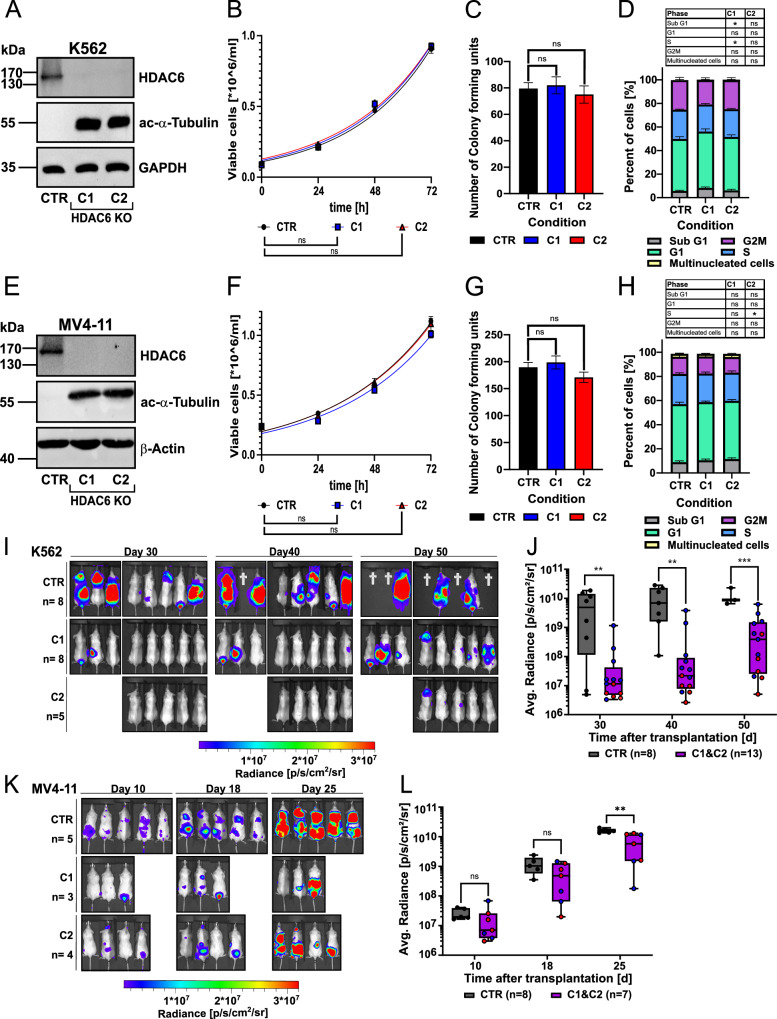
HDAC6 ablation impairs the in vivo growth of myeloid leukemia cells. **A** Representative western blot for HDAC6 and acetylated α-tubulin protein levels in two clones (C1 and C2) of K562 HDAC6-knockout (KO) and a non-targeting control (CTR) (*n* = 3). **B** Proliferation curve determined by trypan blue assay of K562 HDAC6-KO. Statistical significance was calculated by comparing the doubling time of the growth curve (unpaired *t*-test, ns not significant, *n* = 3). **C** Colony-forming unit (CFU) assay performed by seeding K562 HDAC6-KO cells in a semi-solid medium (unpaired *t*-test, ns not significant, *n* = 3). **D** Bar plot showing cell cycle of K562 HDAC6-KO analysis (unpaired *t*-test, ns not significant, *n* = 3). Similarly, analysis was performed in the MV4-11 HDAC6-KO model, including western blot (**E**), proliferation assay (**F**), CFU assay (**G**), and cell cycle analysis (**H**) (unpaired *t*-test, ns not significant, *p < 0.05, *n* = 3). **I**–**K** In vivo growth kinetics of K562 (**I**) and MV4-11 (**K**) leukemia cells in NSG mice, monitored at the indicated time points post-injection using bioluminescence imaging. **J**–**L** Quantification of the average radiance of (**I**–**K**) for K562 (**J**) and MV4-11 (**L**) models (Mann–Whitney *U*-test, ns not significant, *p < 0.05, **p < 0.01, ***p < 0.001).

To address the observed discrepancies between the in vitro and in vivo effects of HDAC6 inhibition, we generated CRISPR-Cas9-mediated HDAC6-knockout (KO) models. Specifically, HDAC6 was deleted in (*KMT2A*-rearranged) MV4-11 AML cells and, in parallel, in (*BCR::ABL1*⁺) K562 chronic myeloid leukemia (CML) cells, given prior reports identifying HDAC6 as a key regulator in CML biology [22, 24, 25], to investigate HDAC6 function across distinct myeloid contexts (Fig. 1A–H). Loss of HDAC6 resulted in increased α-tubulin acetylation, confirming functional impairment of HDAC6 in these models (Fig. 1A, E). First, to evaluate the differences in the cellular growth, a proliferation assays were performed, which revealed no significant differences in doubling time between HDAC6-KO and control cells (Fig. 1B, F). Similarly, a colony formation assay showed no alterations in clonogenic potential upon HDAC6 loss (Fig. 1C, G). Cell cycle analysis further demonstrated minimal changes in the distribution of cells across different phases (Fig. 1D, H). However, we observed an increased susceptibility of K562 HDAC6-KO cells to NK cell-mediated killing in an ex vivo PBMC-derived NK cell killing assay, compared to the respective controls (Supplementary Fig. S1J). Notably, in an in vivo setting, HDAC6-KO (K562 and MV4-11) leukemia models exhibited a significant (*p* < 0.05) reduction in leukemia progression when transplanted into NSG mice compared to controls (Fig. 1I–L). This led to a significant (*p* < 0.01) increase in overall survival in mice injected with HDAC6-KO K562 cells, while no significant differences in survival were observed in the MV4-11 HDAC6-KO model. (Supplementary Fig. S1K–L). This discrepancy in survival outcomes may be attributed to the more aggressive disease kinetics of AML-derived MV4-11 cells relative to CML-derived K562 cells, potentially diminishing the observable survival benefit at later stages despite early and significant reductions in leukemic burden. Taken together, our data, supported by publicly available datasets, suggest that while HDAC6 loss has minimal impact on in vitro proliferation, it significantly impairs the progression of myeloid leukemia cells in vivo.

### Targeting HDAC6 upregulates lysosomal-related protein accumulation

**Fig. 2:**
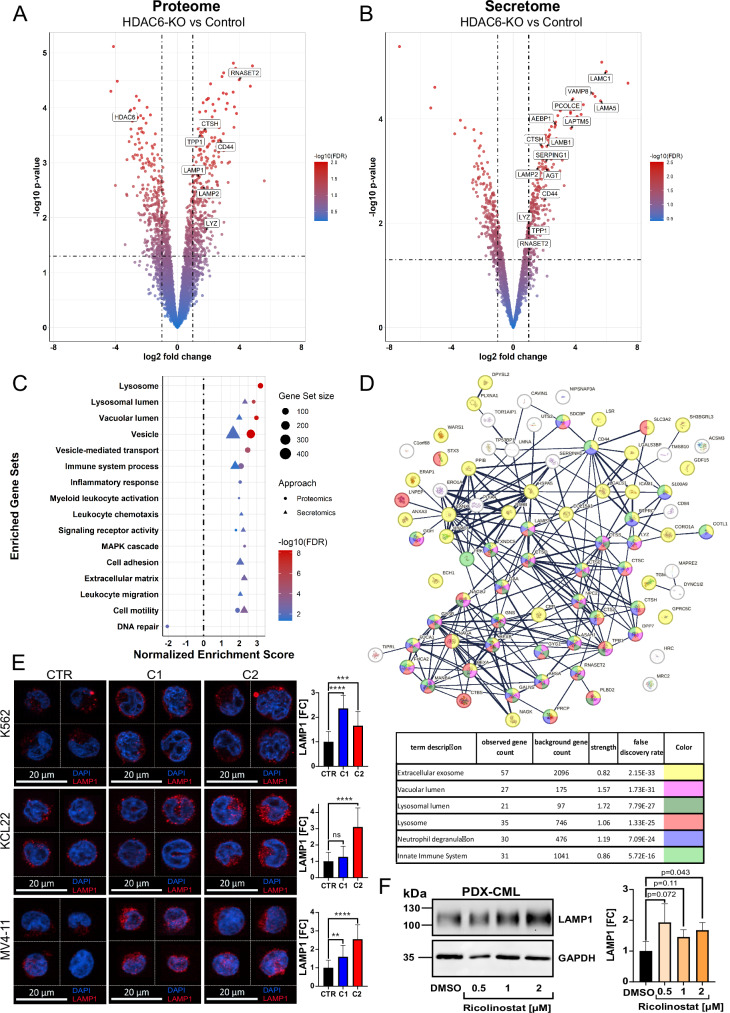
Targeting HDAC6 upregulates lysosome-related protein accumulation. Volcano plot depicting the cellular (**A**) or secreted (**B**) protein log2 fold change against the negative log_10_(*p*-value) determined from five independent replicates of K562 HDAC6-KO (C1). Cut-off for the log2 fold change was set to >1, while for the −log_10_(*p*-value) >1.301 (equals <0.05) was used. The color code represents the −log_10_(FDR) (Significance analysis of microarrays or SAM method, *n* = 4–5). **C** Dot plot showing the manually curated enriched gene sets obtained by a *clusterprofiler* analysis for both proteome and secretome results. The FDR cut-off for inclusion of protein entries was set to < 0.1 (Kolmogorov–Smirnov test, Benjamini–Hochberg correction). **D** Protein interaction network of the overlapping proteins that were enriched both in the proteomics and secretomics analysis via string. Inclusion criteria were a shared positive direction of enrichment and an FDR < 0.1 (hypergeometric test, Benjamini–Hochberg correction). **E** Fluorescence microscopy analyzing the localization and amount of LAMP1 in K562, KCL22, and MV4-11 HDAC6-KO models. Representative cells are shown, while in the right panel, the bar graph compares the fold change of fluorescence signal across all analyzed cells (unpaired *t*-test, ns not significant, **p < 0.01, ***p < 0.001, ****p < 0.0001, *n* = 20 cells). **F** Western blot analysis of LAMP1 and GAPDH expression of PDX-CML cells treated with Ricolinostat, and in the right panel, quantification results normalized to GAPDH (unpaired *t*-test, *n* = 3).

Building on the impaired leukemia progression observed upon HDAC6 loss, we employed quantitative mass spectrometry (MS) to profile global changes in the cellular proteome and secretome (Fig. 2A, B). Gene set enrichment analysis from the proteome analysis identified multiple dysregulated functional pathways upon HDAC6 loss in K562 cells (Fig. 2C and Supplementary Fig. S2A, B). Among the most significantly (*FDR* < 0.05) enriched functional clusters in the proteomic analysis were lysosome-associated proteins, including LAMP1, LAMP2, and RNase T2. Secretome analysis further validated an upregulation of lysosomal enzymes, suggesting increased extracellular release of lysosomal content (Fig. 2B). Additionally, HDAC6-KO cells exhibited enrichment of pathways related to immune activation, inflammatory responses, MAPK signaling and cellular motility (Fig. 2C). Functional enrichment analysis using STRING confirmed significant (*FDR* < 1.E-15) changes in lysosomal and immune-related pathways, including neutrophil degranulation and innate immune system processes, among the overlapping proteins between the proteome and secretome profiles (Fig. 2D and Supplementary Fig. S2C, D). Using fluorescence microscopy, a significant (*p* < 0.01) increase in LAMP1 expression was validated across HDAC6-KO generated models, including K562, (CML-derived) KCL22, and MV4-11 (Fig. 2E and Supplementary Fig. S2E). In addition, HDAC6-KO K562 and MV4-11 cells showed elevated LAMP2 expression (Supplementary Fig. S2F). We further evaluated the lysosomal protease Cathepsin H (CTSH) in the MV4-11 HDAC6-KO models; however, CTSH levels were not significantly elevated upon HDAC6 loss relative to the corresponding control cells (Supplementary Fig. S2G).

To evaluate whether HDAC6 targeting consistently influences the expression of lysosomal proteins in primary disease models, we incorporated (*BCR::ABL1*^+^) PDX-CML cells into our experimental framework. While HDAC6-KO was achievable in established leukemia cell lines, it could not be performed in PDX cells due to their inability to withstand the prolonged culture required for clonal editing. Accordingly, HDAC6 dependency in PDX-CML cells was assessed using short-term pharmacological inhibition with the HDAC6 inhibitor Ricolinostat [33, 34]. Short-term treatment with Ricolinostat also resulted in a concentration-dependent increase in the LAMP1 expression in the PDX-CML cells (Fig. 2F). Together, these results demonstrate that targeting HDAC6 inhibition impacts lysosomal and immune-related pathways.

### HDAC6 loss enhances the accessibility at the LAMP1 and RNase T2 gene loci

**Fig. 3:**
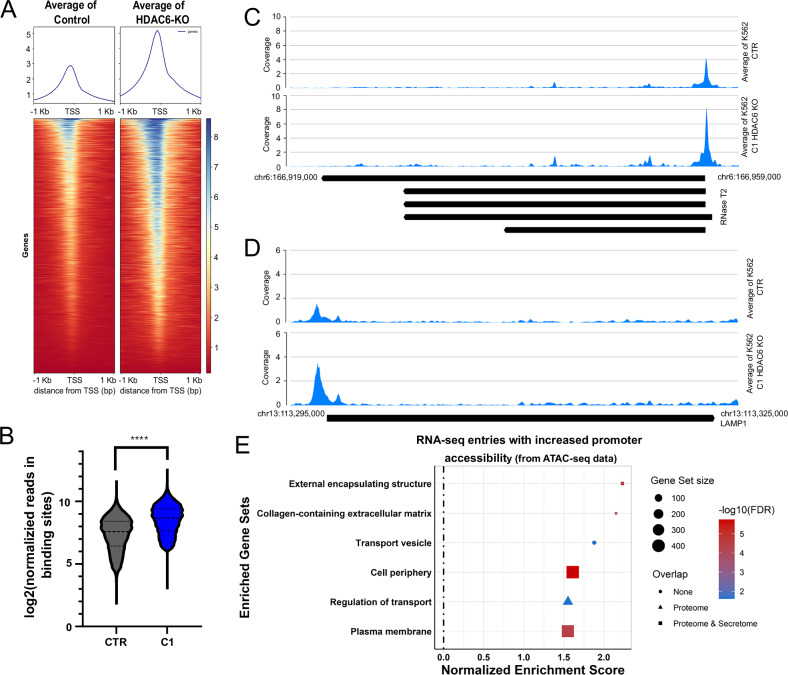
HDAC6 inhibition enhances the accessibility at the LAMP1 and RNase T2 gene loci. **A** Heatmap illustrating the distribution of chromatin accessibility relative to the transcription start site (TSS) in control (CTR) and HDAC6-KO (C1) K562 cells, as determined by ATAC-seq (*n* = 2). **B** Violin plot comparing the log_2_-normalized read density at the binding sites of all significantly enriched genes between CTR and HDAC6-KO (Mann–Whitney *U*-test, ****p < 0.0001, *n* = 10,577). ATAC-seq track plots illustrating chromatin accessibility at the RNase T2 (**C**) and LAMP1 (**D**) loci in CTR and HDAC6-KO K562 cells. **E** Dot plot showing the manually curated enriched gene sets obtained by a *clusterprofiler* analysis for the significant RNA-seq entries with significantly increased accessibility in the promoter region (FDR < 0.05). The shape of the dot indicates whether the term was also significantly upregulated in the Proteome or Secretome (Kolmogorov–Smirnov test, Benjamini–Hochberg correction).

Although HDAC6 primarily resides in the cytoplasm, it can localize to the nucleus and participate in chromatin regulation [35]. To examine genome-wide changes in chromatin accessibility associated with HDAC6 loss, we performed ATAC-seq analysis in K562 HDAC6-KO cells (Fig. 3A, B). In total, 10,577 promoter-associated ATAC-seq peaks were identified, and their accessibility is depicted in the violin plot shown in Fig. 3B. While only a limited number of genomic loci exhibited chromatin compaction following HDAC6 loss, a substantial number of loci and gene sets demonstrated increased accessibility (Supplementary Fig. S3A, B), including loci encoding LAMP1 and RNase T2 (Fig. 3C, D). Next, to assess how increased chromatin accessibility influences global gene expression, we performed RNA-seq on HDAC6-KO (K562) cells (Supplementary Fig. S3C). Gene set enrichment analysis (GSEA) on the RNA-seq data identified upregulation of pathways related to protein acetylation regulation, exocytotic vesicle secretion, and MAPK signaling, while processes associated with HDAC6 function, including aggresome and autophagosome formation, were downregulated (Supplementary Fig. S3D, E) [36, 37]. To integrate our findings, RNA-seq data were overlaid onto the ATAC-seq dataset, revealing a substantial (24.2%) overlap between differentially accessible chromatin regions and transcriptionally upregulated genes (Supplementary Fig. S3F, G). Furthermore, integrated multi-omics analysis, incorporating ATAC-seq, RNA-seq, proteomic, and secretomic data, revealed significant enrichment of gene sets associated with the cell periphery and plasma membrane (Fig. 3E and Supplementary Fig. S3H). These findings suggest that HDAC6 inhibition may regulate surface receptor expression and influence leukemia cell interactions with the surrounding microenvironment [38].

### Targeting HDAC6 induces the expression of tumor suppressor RNase T2 in myeloid leukemia cells

Next, we selected the lysosomal lumen-associated endoribonuclease RNase T2 as a potential target downstream of HDAC6, based on its well-established role as a tumor suppressor [39, 40]. Beyond its enzymatic activity, RNase T2 can trigger immune responses within the tumor microenvironment, particularly by promoting the recruitment of M1-polarized macrophages [41, 42].

**Fig. 4:**
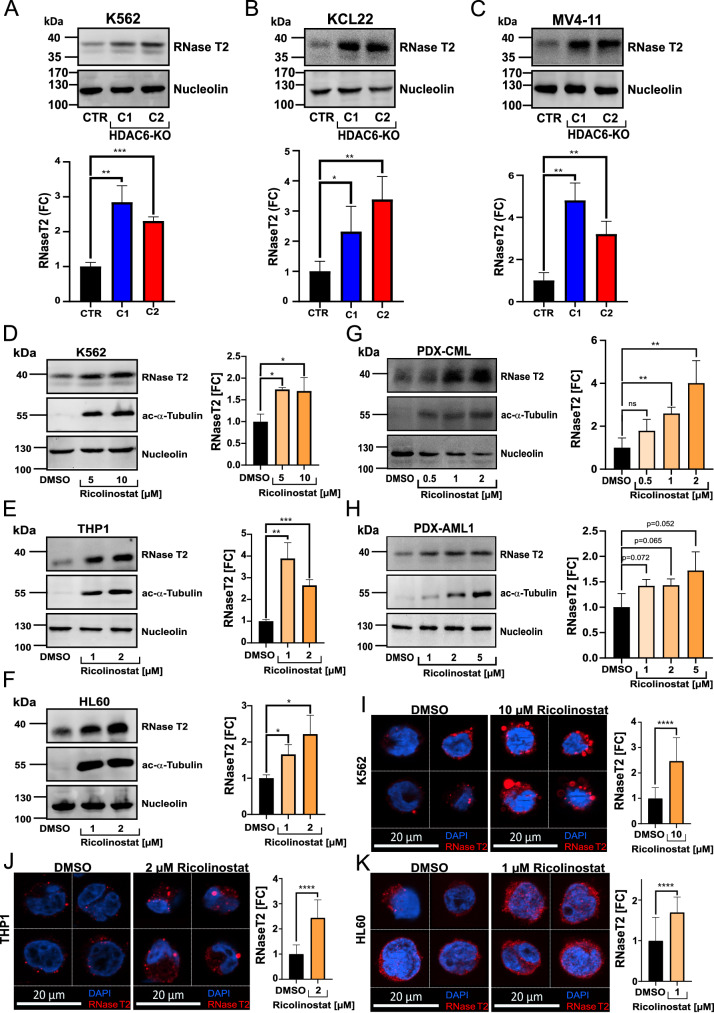
Targeting HDAC6 induces the expression of tumor suppressor RNase T2 in myeloid leukemia cells. Western blot analysis of RNase T2 expression in HDAC6-KO myeloid leukemia models: K562 (**A**), KCL22 (**B**), and MV4-11 (**C**), and in the lower panel, quantification results in the form of a bar graph (unpaired *t*-test, **p < 0.01, ***p < 0.001, *n* = 3). Western blot analysis of RNase T2 and acetylated α-tubulin levels in K562 (**D**), THP-1 (**E**), HL60 (**F**), PDX-CML (**G**), and PDX-AML1 (**H**) cells following 24 h of treatment, or 48 h for HL60, with the HDAC6 inhibitor Ricolinostat at the indicated concentrations. The right panel shows the results of the quantification normalized to housekeeper Nucleolin (unpaired *t*-test, *p < 0.05, **p < 0.01, ***p < 0.001, *n* = 3). Fluorescence microscopy analysis of RNase T2 localization and expression in K562 (**I**), THP-1 (**J**), and HL60 (**K**) cells treated with Ricolinostat for 24 h at the indicated concentrations. Representative cells are shown, while in the right panel, the bar graph compares the fold change of fluorescence signal across all analyzed (*n* = 20) cells (unpaired *t*-test, ns not significant, ****p < 0.0001).

Notably, all HDAC6-KO models (K562, KCL22, and MV4-11) demonstrated a consistent and significant (*p* < 0.05) upregulation of RNase T2 (Fig. 4A–C). Consistent with the MS-based secretome analysis, HDAC6-KO K562 cells exhibited elevated levels of RNase T2 in the culture supernatant (Supplementary Fig. S4A). Next, to determine whether RNase T2 upregulation upon HDAC6 inhibition is reversible, we performed knock-in (KI) experiments by reintroducing HDAC6 into HDAC6-KO (K562) cells. Functional validation of HDAC6 re-expression was confirmed by the reversal of α-tubulin hyperacetylation (Supplementary Fig. 4B). HDAC6 restoration in HDAC6-KO cells also led to a significant (*p* < 0.05) reduction in RNase T2 expression, supporting the role of HDAC6 in regulating RNase T2 expression.

Next, to determine whether this effect was specific to myeloid leukemia cells or extended to ALL cells, we assessed RNase T2 protein levels in HDAC6-KO or knockdown (KD) models using B-cell acute lymphoblastic leukemia (B-ALL) cell lines (NALM6 and NALM20) and in (*BCR::ABL1*^*+*^) PDX-B-ALL2 cells (Supplementary Fig. S4C–F and Supplementary Table 3 for PDX information). Specifically, RNase T2 expression remained unchanged or even downregulated upon HDAC6 loss in the tested B-ALL models. Consistently, correlation analysis (using the St. Jude PeCan database) revealed that in myeloid leukemia patients, HDAC6 and RNase T2 expression levels are negatively correlated (*r* = −0.13, *p* < 0.05), whereas in lymphoid leukemia patients, a strong positive correlation (*r* = 0.53, *p* < 0.0001) was observed (Supplementary Fig. S4G–J).

Furthermore, short-term treatment with the HDAC6 inhibitor Ricolinostat at sub-cytotoxic concentrations (selected based on the differential sensitivity of each cell model and validated by increased acetylated α-tubulin levels as a readout of HDAC6 inhibition) resulted in a significant (*p* < 0.05) upregulation of RNase T2 across multiple myeloid leukemia cell lines, including K562, THP-1, and HL60 (Fig. 4D–F). Additionally, we included (*BCR::ABL1*^+^) PDX-CML and (*RUNX1-*mutated) PDX-AML1 cells in the assay, which also revealed a concentration-dependent increase in RNase T2 levels upon treatment with Ricolinostat (Fig. 4G, H). Immunofluorescence microscopy further confirmed that RNase T2 accumulated in a punctate pattern upon Ricolinostat treatment, in myeloid leukemia (K562, MV4-11, THP-1, and HL60) cells (Fig. 4I–K and Supplementary Fig. S4K). Consistently, short-term treatment with Ricolinostat or the other HDAC6 inhibitor Citarinostat resulted in increased RNase T2 transcription in AML-derived (*KMT2A*r) THP-1 cells, as well as in (*BCR::ABL1*^*+*^) PDX-CML and (*KMT2A*r) PDX-AML7 cells (Supplementary Fig. S4L, M).

These findings suggest that HDAC6 inhibition effectively induces a phenotype characterized by a low HDAC6/RNase T2 ratio. Notably, a survival analysis (using the TARGET dataset) showed that AML patients with a low HDAC6/RNase T2 ratio had significantly (*p* = 0.0003) improved survival outcomes in comparison to those with a high ratio, whereas the opposite trend was observed in the ALL dataset. (Supplementary Fig. S4N, O). Additionally, better survival in AML was associated with lower levels of anti-inflammatory monocytes and M2 macrophages (Supplementary Fig. S4P, Q). Taken together, HDAC6 regulates RNase T2 expression in myeloid leukemia cells.

### HDAC6 inhibition sensitizes myeloid leukemia cells to CD8^+^ T cells

**Fig. 5:**
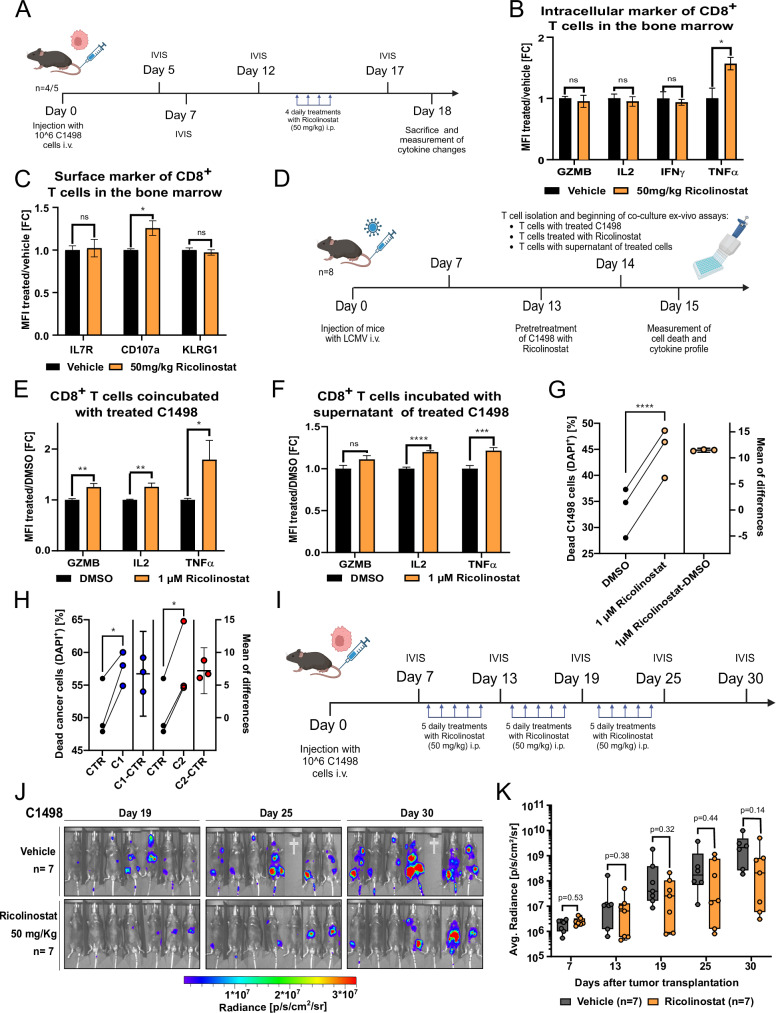
HDAC6 inhibition sensitizes myeloid leukemia cells to CD8^+^ T cells. **A** Experimental timeline of Ricolinostat (50 mg/kg) treatment following the injection of murine C1498-Luc-GFP + AML cells into wild-type C57BL/6 mice to determine CD8+ T cell marker level. Bar graph comparing the fold change in mean fluorescence intensity (MFI) of intracellular (**B**) or surface (**C**) marker of CD8^+^ T cells from the bone marrow after Ricolinostat treatment against the control (unpaired *t*-test, ns not significant, *p < 0.05, *n* = 4–5). **D** Experimental timeline for the harvest of primed CD8^+^ T cells from C57BL/6 mice following Lymphocytic Choriomeningitis Virus (LCMV) injection, followed by ex vivo co-culture with C1498 cells. Bar graph showing the fold change in MFI of quantified cytokines from CD8^+^ T cells co-incubated with C1498 cells treated for 24 h with 1 µM Ricolinostat (**E**) or the supernatant from Ricolinostat-treated C1498 cells (**F**) (unpaired *t*-test, *p < 0.05, **p < 0.01, ***p < 0.001, ****p < 0.0001, *n* = 16). **G** Line plot showing the fraction of dead C1498 cells when co-incubated with CD8^+^ T cells, pretreated with Ricolinostat or DMSO (paired *t*-test, ****p < 0.0001, *n* = 3). **H** Similar to (**G**), but using C1498 HDAC6-KO cells (in two clones, C1 or C2) vs non-targeting control (CTR). **I** Experimental timeline of Ricolinostat (50 mg/kg) treatment following the injection of murine C1498-Luc-GFP + AML cells into wild-type C57BL/6 mice to determine disease progression. **J** In vivo growth kinetics of C1498 cells in C57BL/6 mice treated with Ricolinostat, assessed at 19-, 25-, and 30-days post-injection via luminescence detection. **K** Quantification of the average radiance from (**J**) (Mann–Whitney *U*-test, *n* = 7).

Based on our multi-omics and in vivo data suggesting that targeting HDAC6 affects immune-related signaling, we aimed to further explore its immunomodulatory role in a more immunocompetent in vivo setting. We therefore employed a syngeneic mouse model using C1498 cells, derived from C57BL/6 mice and classified as acute myelomonocytic leukemia [43]. Briefly, murine C1498-Luciferase-GFP+ cells were injected into immunocompetent C57BL/6 wild-type mice (Fig. 5A). After confirming engraftment, the mice were treated with four doses of Ricolinostat (50 mg/kg). Bone marrow, spleen, and lymph nodes were collected after the last treatment. A significant increase (*p* < 0.05) in TNFα production was observed in bone marrow-infiltrating T cells from Ricolinostat-treated mice, whereas only a modest (non-significant) elevation was detected in splenic T cells, and no changes were observed in lymph node T cells (Fig. 5B and Supplementary Fig. S5A, B). Similarly, CD107a (LAMP1) expression was upregulated in bone marrow T cells and unchanged in spleen- and lymph node-derived T cells (Fig. 5C and Supplementary Fig. S5C, D), a marker of CTL lytic granule release involved in target-cell killing [44].

To validate these findings, an ex vivo co-culture model was established with syngeneic murine CD8^+^ T cells (Fig. 5D). Treatment of C1498 cells with Ricolinostat induced a significant (*p* < 0.01) upregulation of GZMB, IL-2, and notably TNFα in CD8^+^ T cells (Fig. 5E). Moreover, the supernatant from Ricolinostat-treated C1498 cells was enough to elicit the same response in T cells, while Ricolinostat treatment alone did not significantly affect the expression of these markers (Fig. 5F and Supplementary Fig. S5E). Notably, treatment with Ricolinostat consistently enhanced CD8^+^ T cell-mediated cytotoxicity of C1498 cells (Fig. 5G). Consistent with observations in human myeloid leukemia cells, Ricolinostat treatment led to an upregulation of RNase T2 expression in murine C1498 cells (Supplementary Fig. S5F).

To further validate the involvement of HDAC6 in eliciting T cell responses, a C1498 HDAC6-KO model was generated (Supplementary Fig. S5G). While the cytokine response was less pronounced with the C1498 HDAC6-KO model, we still observed a significant (*p* < 0.05) increase in CD8^+^ T cell susceptibility to cytotoxicity and cytokine responses, similar to the (wild-type) C1498 cells upon Ricolinostat treatment (Fig. 5H and Supplementary Fig. S5H, I). Next, to assess the therapeutic benefit of Ricolinostat as a monotherapy, we treated C57BL/6J mice following engraftment with C1498-Luc-GFP+ cells over a prolonged period (3 weeks) and monitored leukemia progression (Fig. 5I). Although Ricolinostat treatment showed a trend toward restricting leukemia progression, the results were not statistically significant, indicating that while HDAC6 inhibition enhances T cell-mediated immune responses (Fig. 5J, K), and its therapeutic benefit in vivo requires additional combination partners.

### Targeting HDAC6 sensitizes myeloid leukemia cells toward standard chemotherapeutics, Cytarabine and Clofarabine

**Fig. 6:**
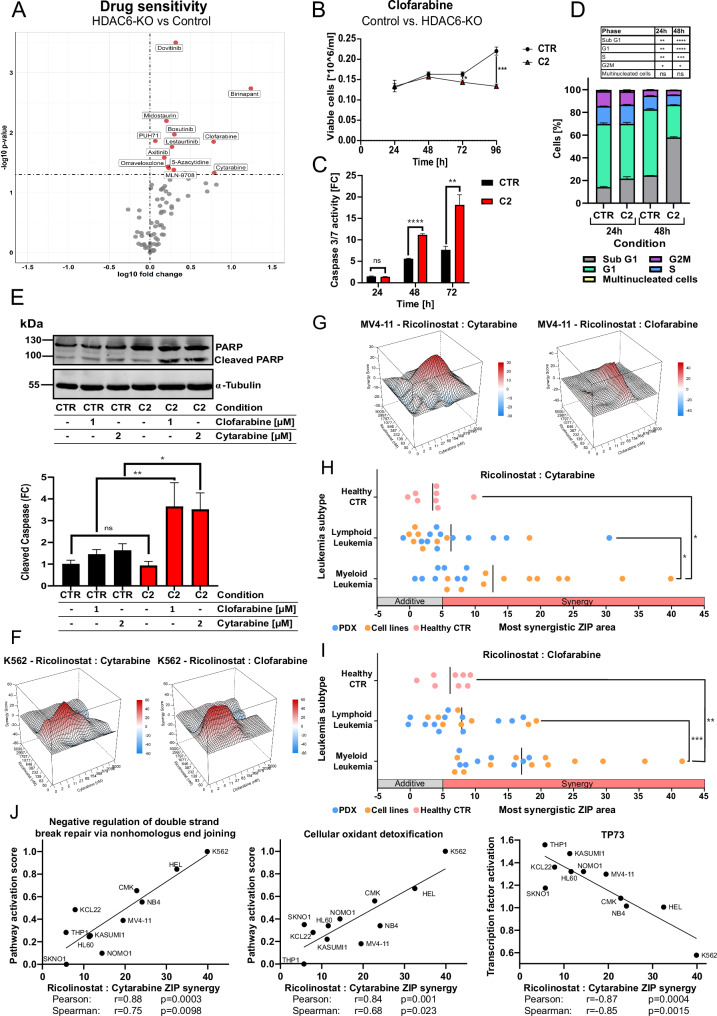
Targeting HDAC6 sensitizes myeloid leukemia cells toward standard chemotherapeutics Cytarabine and Clofarabine. **A** Volcano plot depicting the log_10_(IC50 FC) and the –log_10_(p-value) between K562 CTR and K562 HDAC6-KO (C1 and C2). As cut-off was −log_10_(*p*-value) >1.301 (equals <0.05) used (unpaired *t*-test, *n* = 3–6). **B** Proliferation curve determined by trypan blue assay of K562 CTR and K562 HDAC6-KO C2 treated with 1 µM Clofarabine over the course of 96 h (unpaired *t*-test, *p < 0.05, ***p < 0.001, *n* = 3). **C** Bar plot of the fold change of the caspase 3/7 activity of K562 CTR and K562 HDAC6-KO C2 treated with 1 µM Clofarabine over the course of 72 h (unpaired *t*-test, ns not significant, **p < 0.05, ****p < 0.0001, *n* = 3). **D** Bar plot showing a cell cycle analysis of K562 HDAC6-KO models treated with Clofarabine. Statistical significance was determined via an unpaired *t*-test (ns not significant, *p < 0.05, **p < 0.01, ***p < 0.001, ****p < 0.0001, *n* = 3). **E** Western Blot analysis of PARP and cleaved PARP of K562 HDAC6-KO models treated with 1 µM Clofarabine or 2 µM Cytarabine. The lower panel bar graph shows the results of the quantification of cleaved PARP normalized to the housekeeper α-tubulin (unpaired *t*-test, *p < 0.05, **p < 0.01, *n* = 3). Matrix synergy plots for two leukemia cell lines. In K562 cells (**F**) and MV4-11 cells (**G**), the plots show the effects of increasing concentrations of Ricolinostat combined with increasing concentrations of Cytarabine or Clofarabine. Zero interaction potency (ZIP scores) indicate synergy for the pairings: Ricolinostat:Cytarabine in K562 (39.9) and MV4-11 (19.5); Ricolinostat:Clofarabine in K562 (41.6) and MV4-11 (21.1). Dot plot summarizing the most synergistic ZIP scores of AML/CML cell lines (*n* = 12), patient-derived PDX-AML/CML cells (*n* = 8), ALL cell lines (*n* = 8), PDX-ALLs (*n* = 12), and healthy controls (*n* = 8), when treated with Ricolinostat and Cytarabine (**H**) or Ricolinostat and Clofarabine (**I**) combination. Statistical significance was determined using an unpaired *t*-test (ns not significant, *p < 0.05, **p < 0.01, ***p < 0.001). **J** Pathway and transcription factor activities were inferred from gene expression data of AML and CML cell lines (from DepMap portal, 25Q2) and correlated with ZIP synergy scores of the Ricolinostat and Cytarabine combination.

To enhance the therapeutic impact of HDAC6 inhibition and identify potential anti-cancer combination partners, we conducted a synthetic lethality screen using HDAC6-KO (K562) cells (Fig. 6A). HDAC6-KO cells showed significantly (*p* < 0.05) increased sensitivity to several drug classes, including tyrosine kinase inhibitors (TKIs) and anti-metabolites such as Clofarabine, Cytarabine, and 5-Azacytidine. Given the clinical relevance of Cytarabine in treating AML, we examined its effects alongside Clofarabine. Both drugs exhibited enhanced cytotoxicity in HDAC6-KO cells, with Clofarabine showing a particularly strong reduction in proliferation (Fig. 6B and Supplementary Fig. S6A). HDAC6-KO cells also displayed increased apoptosis induction (*p* < 0.01) and sub-G1 accumulation following treatment with Cytarabine or Clofarabine (Fig. 6C, D, and Supplementary Fig. S6B, C). Elevated levels of PARP cleavage (*p* < 0.05) following treatment with Cytarabine or Clofarabine further confirmed enhanced apoptotic responses in HDAC6-KO cells compared to corresponding controls (Fig. 6E). These findings suggest that HDAC6 loss can enhance the sensitivity to nucleoside analogs.

To validate these findings, we investigated potential synergistic interactions between Ricolinostat and Cytarabine or Clofarabine. Both Cytarabine and Clofarabine exhibited strong synergistic effects with Ricolinostat in K562 and MV4-11 cells (Fig. 6F, G). Encouraged by these findings, we extended our investigation to a broader cohort, including (*n* = 10) AML, (*n* = 4) B-ALL, and (*n* = 4) T-ALL cell lines. Additionally, we included PDX-AML/CML cells derived from initial or relapsed pediatric/adult patients (*n* = 8), along with PDX-B-ALL (*n* = 5), PDX-T-ALL (*n* = 7), and healthy donor-derived PBMCs (*n* = 4), fibroblasts (*n* = 2), mesenchymal stem cells (MSCs), and cord blood-derived CD34⁺ cells as controls (Fig. 6H, I, and Supplementary Fig. S6D, E). Notably, myeloid lineage-derived leukemia cells demonstrated heightened sensitivity to the HDAC6 inhibitor Ricolinostat, when combined with Clofarabine or Cytarabine, compared to B-ALL, T-ALL, and healthy control samples. Next, to investigate mechanisms associated with the observed synergy between HDAC6 inhibitor (Ricolinostat) and Clofarabine or Cytarabine, we correlated publicly available transcriptomic profiles (from DepMap portal, 25Q2) of AML and CML cell lines with experimentally determined (ZIP) synergy scores (Fig. 6J and Supplementary Fig. S6F). This analysis revealed a positive correlation between synergistic response and gene sets involved in the negative regulation of DNA double-strand break repair via non-homologous end joining, as well as cellular oxidant detoxification pathways. These findings suggest that impaired DNA repair capacity and elevated oxidative stress may enhance the efficacy of combination therapy. Conversely, a negative correlation was observed with TP73-mediated transcription factor activation, a member of the p53 family involved in DNA damage response and apoptosis regulation. To experimentally interrogate these findings, HDAC6-KO (K562 and MV4-11) cells were treated with Cytarabine and Clofarabine (Supplementary Fig. 6G, H). In both models, HDAC6 ablation resulted in a pronounced induction of TP73 protein levels relative to control cells, consistent with activation of a compensatory DNA damage response upon genotoxic stress [45, 46]. Together, these data point to a potential mechanistic link between HDAC6 inhibition and DNA damage response to enhance chemotherapeutic efficacy upon HDAC6 inhibition in myeloid leukemias.

## Discussion

Despite substantial progress in elucidating the molecular pathogenesis of AML, therapeutic strategies have remained largely unchanged, relying predominantly on conventional chemotherapy and allogeneic hematopoietic stem-cell transplantation [3, 47–49]. Therefore, there is a pressing need to identify and develop novel therapeutic strategies. HDAC6 has emerged as a promising anti-cancer target [15]. The present study reveals a critical discrepancy between the in vitro and in vivo functions of HDAC6 in myeloid leukemia, challenging the prevailing understanding of HDAC6’s role in hematologic malignancies. While previous ex vivo studies have demonstrated minimal cytotoxic effects of selective HDAC6 inhibition on cancer cells [28–30], our comprehensive analysis using public datasets, CRISPR-mediated knockout models, and in vivo xenograft studies provides compelling evidence that HDAC6 plays a significant role in (myeloid) leukemia progression within the physiological context of the tumor microenvironment. The absence of significant differences in proliferation rates, colony formation capacity, and cell cycle distribution between HDAC6-KO and control cells in vitro corroborates previous findings and explains why ex vivo screening approaches (like DepMap Portal) may have overlooked HDAC6 as a therapeutic target. However, the significant reduction in leukemia progression observed in NSG mice transplanted with HDAC6-KO leukemia cells demonstrates that HDAC6’s essential functions become apparent only within the complex in vivo environment, potentially via modulation of innate immune pathways that remain active in the NSG mice model [50].

Our proteomic analyses reveal that HDAC6 loss results in significant enrichment of lysosome-associated proteins, including LAMP1, LAMP2, and RNase T2, consistent with HDAC6’s established role in coordinating aggresome transport and autophagosome-lysosome fusion through cortactin and cytoskeletal interactions [51]. HDAC6 facilitates lysosomal transport along microtubules via dynein-mediated mechanisms, enabling lysosome-autophagosome co-localization and fusion [52, 53]. Loss of HDAC6 can therefore lead to lysosomal accumulation, as reported in this study showing increased LAMP2 levels and reduced autophagic activity upon HDAC6 knockdown [54]. The disruption of these interconnected pathways upon HDAC6 loss may contribute to altered lysosomal protein expression and secretion, where lysosomal accumulation could arise from both impaired trafficking and compensatory biogenesis. Further, our ATAC-seq analyses demonstrate increased chromatin accessibility at LAMP1 and RNase T2 loci, potentially providing a supplementary transcriptional mechanism for lysosomal protein upregulation. However, we did not observe a significant increase in the lysosomal protease CTSH in MV4-11 HDAC6-KO cells, as seen in contrast to proteomics studies in K562 cells, indicating that HDAC6 loss exerts selective rather than uniformly affecting all lysosomal components, which may vary across genetically distinct myeloid leukemia contexts. Importantly, the extracellular release of lysosomal enzymes, as evidenced by secretome analysis, could potentially alter the local microenvironment and enhance immune cell function. Supporting this, our STRING analysis revealed enrichment of neutrophil degranulation and innate immune pathways among overlapping proteome-secretome proteins, indicating potential immunomodulatory effects of HDAC6 loss [55]. Collectively, the integrated multi-omics analysis reveals enrichment of cell periphery and plasma membrane-associated gene sets, suggesting that HDAC6 inhibition may modulate surface receptor expression and leukemia-microenvironment interactions.

RNase T2, a lysosomal endonuclease and evolutionarily conserved component of the innate immune system that functions as an alarmin-like molecule within the tumor microenvironment to promote macrophage recruitment and pro-inflammatory activation [41, 42], emerges as a novel mediator in the anti-leukemic effects of HDAC6 targeting. We observed consistent upregulation of RNase T2 across all HDAC6-KO myeloid leukemia models, coupled with its elevated secretion into the extracellular environment, suggesting that HDAC6 normally functions to suppress this tumor suppressor. The reversibility of RNase T2 upregulation upon HDAC6 reintroduction in knock-in experiments confirms the direct regulatory relationship between these proteins. Notably, this regulatory axis is lineage-specific, as evidenced by differential responses observed when modeling HDAC6 loss or knockdown in ALL versus AML cells, which was further supported by distinct correlation patterns between HDAC6 and RNase T2 expression in myeloid versus lymphoid leukemias from public datasets. Supporting this, survival analysis revealed that AML patients with a low HDAC6/RNase T2 ratio had significantly improved survival compared to those with a high ratio, with the opposite trend observed in ALL patients. Additionally, a better survival in AML was associated with lower levels of anti-inflammatory monocytes and M2 macrophages, consistent with previous findings where HDAC6 inhibition or RNase T2 upregulation promotes macrophage polarization toward a pro-inflammatory phenotype [40, 56]. However, the molecular basis underlying this lineage-specificity remains unclear and warrants further investigation to elucidate whether differential epigenetic landscapes or lineage-restricted cofactors contribute to the divergent HDAC6-RNase T2 regulatory dynamics between myeloid and lymphoid leukemias. Further, pharmacological validation with Ricolinostat across myeloid leukemia cell lines and PDX models supports the translational relevance of the HDAC6-RNase T2 axis. We also observed increased RNase T2 mRNA levels in PDX-AML/CML samples upon Ricolinostat treatment, although the magnitude of induction varied, likely reflecting underlying genetic and epigenetic heterogeneity among individual samples. However, it is important to note that Ricolinostat is not an HDAC6-selective inhibitor, and its effects may also result from off-target actions or inhibition of other HDACs, making it difficult to attribute all observed effects solely to HDAC6 inhibition [57, 58]. Accordingly, these findings should be interpreted with caution. To ensure that the observed effects were attributable to on-target HDAC6 inhibition rather than compound-specific artifacts, we included another HDAC6 inhibitor, Citarinostat, as an orthogonal pharmacological validation. Nevertheless, additional validation using highly selective HDAC6 inhibitors, together with acetylation readouts of proteins not regulated by HDAC6, would strengthen the specificity controls and help confirm whether the observed regulation of RNase T2, and the associated anti-leukemic effects, are indeed HDAC6-dependent.

Extending our findings to immunocompetent models, HDAC6 inhibition enhanced CD8^+^ T cell activation [59], through TNFα induction and upregulation of CD107a (or LAMP1). The significant increase in TNFα production and CD107a expression specifically in bone marrow-infiltrating T cells from Ricolinostat-treated mice indicates that HDAC6 inhibition enhances T cell activation and cytolytic function in the primary site of leukemia development. Consistently, we have observed an increase in lysosome-associated proteins upon targeting HDAC6, which aligns with emerging evidence that the lysosome plays a crucial role in regulating the cytotoxic activity of CD8^+^ T cell immunity [60]. Moreover, in our ex vivo co-culture assays, Ricolinostat alone elicited only a modest, non-significant increase in TNF-α in CD8⁺ T cells, indicating limited direct activation. In contrast, markedly stronger cytokine and effector responses were observed when T cells were exposed to Ricolinostat-treated (C1498) leukemia cells or their supernatants, supporting a leukemia cell-mediated mechanism, while not fully excluding direct effects of HDAC6 inhibition on T cells [61, 62]. The enhanced CD8^+^ T cell-mediated cytotoxicity observed upon Ricolinostat treatment represents a potential therapeutic option by which HDAC6 inhibition may improve myeloid leukemia outcomes in vivo. However, while Ricolinostat monotherapy showed a trend toward reduced (C1498) leukemia progression in an immunocompetent mouse model, the lack of statistical significance suggests that HDAC6 inhibition alone is insufficient for therapeutic efficacy and requires combination strategies.

Next, to identify potential therapeutic partners for HDAC6 inhibition, we performed a synthetic lethality screen and validation experiments in HDAC6-KO K562 cells to establish proof-of-concept that loss of HDAC6 sensitizes cells to nucleoside analogs (Cytarabine and Clofarabine). Building on this, pharmacological validation combining Ricolinostat with these agents across multiple AML and ALL models demonstrated stronger and more consistent synergistic effects in AML compared to ALL, with minimal impact on healthy controls. These findings align with prior reports that dual HDAC1/HDAC6 inhibition enhances cytarabine responses in AML [34] and are further supported by our transcriptomics and proteomics showing downregulation of DNA repair pathways in HDAC6-KO cells [45, 46]. Furthermore, we demonstrate that loss of HDAC6 enhances TP73 levels in response to Cytarabine and Clofarabine, which may reflect engagement of a protective, TP73-dependent stress response. This observation is consistent with our pathway analysis, revealing a negative association between TP73-mediated transcriptional programs and synergistic drug responses. However, as these findings were derived from limited cell line models, the mechanistic relationship between HDAC6 inhibition, TP73 activation, and DNA damage response remains preliminary. Future studies should extend these observations across additional myeloid models and incorporate functional assays to directly interrogate DNA repair pathways.

Further analysis of over 1500 leukemia patient samples (from the St. Jude PeCan database) revealed more heterogeneous HDAC6 expression patterns in AML compared to B-ALL and T-ALL (coefficient of variation: AML = 0.70 vs. B-ALL = 0.48 vs. T-ALL = 0.39) (Supplementary Fig. S6I). Given this heterogeneous HDAC6 expression across AML subtypes, future studies should prioritize identifying molecularly defined patient subgroups with enhanced sensitivity to HDAC6 inhibition. Our data mining analyses demonstrated that high HDAC6 expression is particularly associated with poor prognosis in AML cases harboring NRAS mutations, as well as in FLT3- and IDH1-mutated subtypes (Supplementary Fig. S1E–H). These findings suggest that specific genetic contexts may confer differential dependency on HDAC6, warranting further investigation to establish biomarker-driven therapeutic strategies for HDAC6 targeting in genetically stratified AML patient populations. Together, our study provides compelling evidence that HDAC6 inhibition enhances immune-mediated leukemia cell clearance and sensitizes myeloid leukemia cells to chemotherapeutic agents. Given the minimal toxicity associated with HDAC6 inhibition, these findings support its potential use in combination with emerging immunotherapeutic strategies to enhance anti-leukemic efficacy.

## Materials and methods

### Cell culture

Leukemia cell lines were obtained from the DSMZ (Braunschweig, Germany) and cultured according to their recommendations. RPMI1640 GlutaMAX (Gibco) was supplemented with 10–20% fetal bovine serum and 1% penicillin/streptomycin (Sigma-Aldrich). Cell lines were regularly authenticated by short tandem repeat profiling and tested for mycoplasma. A detailed list of cell lines used in the study is provided in Supplementary Table 1.

### CRISPR-Cas9-mediated knockout (KO) or shRNA-mediated knockdown (KD) of HDAC6

HDAC6-KO models were generated either through lentiviral transduction or using nucleofection of CAS9-GFP (IDT) [63]. For monoclonal selection, cells were plated in semi-solid MethoCult media (Stemcell Technologies). See Supplementary Methods for sequences and more details.

### Western blotting

Western blotting was performed as previously described [63]. For the list of antibodies and their respective concentrations, refer to the Supplementary Methods.

### Proliferation, cell cycle, and apoptosis assays

Proliferation (via Trypan Blue staining), cell cycle (via Nicoletti method), and apoptosis assay (via measuring Caspase 3/7 activity) were performed as described earlier [63].

### Colony-forming unit (CFU) assay

Leukemia cells were seeded in a methylcellulose-based medium. After seven days, colonies were counted and imaged, following previously described protocols [64].

### Drug screening

Ex vivo high-throughput drug screening was performed as described earlier [63, 65]. The compound selection comprised FDA/EMA-approved chemotherapeutics and targeted agents commonly used in leukemia treatment protocols, along with inhibitors in various stages of clinical trials. Compounds were dispensed in a randomized layout using a digital dispenser (D300e, Tecan). A detailed list of drugs is provided in Supplementary Table 2. ZIP scores for the matrix synergy approach were calculated via the *Synergyfinder* R package.

### qPCR

Total RNA was extracted using QIAzol and purified with the Maxwell RSC system. cDNA was synthesized from 2 µg RNA using the QuantiTect kit, and qPCR was performed on a Bio-Rad system with B2M and GAPDH as internal controls. For primer sequences and additional details, refer to Supplementary Methods.

### Fluorescence microscopy

Cells were seeded on poly-D-lysine-coated coverslips, fixed, permeabilized, and stained with primary/secondary antibodies and DAPI. Imaging was performed with a Zeiss Axio Observer/Apotome 3, and signal quantification was done using ImageJ. For more details, refer to Supplementary Methods.

### Murine NSG transplantation

Prior to the injection, K562 and MV4-11 cells were transduced with a luciferase reporter plasmid to measure the leukemic burden via the in vivo imaging system IVIS (PerkinElmer) [63]. Cells were intravenously injected into 6–8 weeks old immunodeficient male or female NSG mice (Jackson Laboratory, #005557). For patient-derived xenograft (PDX) generation, leukemia cells from peripheral blood or bone marrow were injected into NSG mice (see Supplementary Table 3 for patient details), with engraftment monitored via FACS analysis of blood collected through orbital bleeding [63, 66]. Patient samples were obtained with informed consent in accordance with the Declaration of Helsinki, with approval from the local ethics committees at the sites in Düsseldorf, Vienna, and Lodz. All animal experiments were conducted in accordance with the regulatory guidelines of the official committee at LANUV (NRW), under the authorization of the animal research institute (ZETT) at the Heinrich Heine University Düsseldorf.

### Murine C57BL/6J experiments

Wildtype male C57BL/6J (Charles River) mice were utilized to establish a murine C1498 AML (ATCC) derived syngeneic mouse model [67]. C57BL/6J wild-type mice were engrafted with (0.5 × 10^6^ cells/mouse) luciferase reporter gene transduced C1498 murine AML cell line at 6–7 weeks of age. Engraftment was confirmed 7 days after injection of C1498 leukemia cells using IVIS (PerkinElmer), and mice were then randomly assigned to treatment groups (*n* = 7 mice/arm). No blinding or randomization was performed. Group sizes were not predefined by statistical calculation and were selected based on previous experiments and pilot data. Monitoring of the leukemic burden and treatment with Ricolinostat followed the respective schedule. For more details, refer to the Supplementary Methods.

### T cell co-culture assay

C57BL/6J mice were infected with the Lymphocytic Choriomeningitis Virus (LCMV) strain, which induces a strong effector CD8⁺ T cell response and is efficiently cleared in wild-type mice shortly after infection [67, 68]. LCMV-primed splenic T cells were isolated after two weeks post infection. C1498 cells were pretreated with Ricolinostat before co-culture with T cells. Supernatant-only and direct treatment conditions were also included. After 24 h, cells were analyzed by flow cytometry for viability and cytokine expression. For more details, refer to Supplementary Methods.

### NK cell killing assay

PBMCs from healthy donors were cultured with IL-2 and IL-15. CFSE-labeled target cells were co-incubated with PBMC-derived NK cells for 4 h, followed by flow cytometric analysis to assess NK cell-mediated cytotoxicity. For more details, refer to the Supplementary Methods.

### RNA-sequencing (RNA-seq), mass spectrometry (MS) based proteome and secretome analysis

RNA-seq and quantitative MS-based proteome and secretome analysis were performed as previously described [63]. For sample preparation, cultures were maintained at a medium density for several days to harmonize viability and growth rates.

### ATAC-seq

The library preparation, sequencing, and initial consensus sequencing were performed according to the protocol of Active Motif. For more details, refer to Supplementary Methods.

### Data mining

The HDAC6 dependency data was extracted from the Achilles gene dependency project of the DepMap (22Q1) database and visualized via a complex heatmap [69]. The expression data of pediatric patients was extracted from the PeCan platform of St. Jude [70]. Expression was either clustered by leukemia subtype or separated by the median of HDAC6 expression. The survival analysis was performed using the Survival Genie 2 platform [71], utilizing data generated by the Therapeutically Applicable Research to Generate Effective Treatments (TARGET) initiative (https://www.cancer.gov/ccg/research/genome-sequencing/target), phs000218 [31]. The data used for this analysis are available through the Genomic Data Commons (https://portal.gdc.cancer.gov). Samples were clustered by their optimal cutting point algorithm. Mutation clustered survival analysis was performed via Kaplan–Meier-plotter [32], which utilizes the TCGA-LAML dataset. Patients with IDH1, NRAS, or FLT3 mutation were split by a percentile-based best cut-off selection.

Pathway activity scores were inferred using Pathifier [72], and transcription factor activity was estimated using VIPER with the DoRothEA regulon [73], based on gene expression data from AML and CML cell lines in the DepMap (25Q2) dataset. The resulting activity profiles were then correlated with ZIP synergy scores of the Ricolinostat and Cytarabine or Clofarabine combination using both Pearson and Spearman correlation coefficients.

### Statistics

Experiments were reproduced at least three times, unless specified otherwise in the respective section. Comparisons between two groups were evaluated using unpaired or paired two-tailed *t*-tests, whereas non-parametric comparisons of in vivo bioluminescence data were assessed using the Mann–Whitney *U*-test. Kaplan–Meier survival curves for patient datasets and mouse studies were analyzed using the log-rank test. For multi-omics datasets, significance thresholds were defined by false discovery rate (FDR) correction using the Benjamini–Hochberg method, including Significance Analysis of Microarrays (SAM) method for proteomics and secretomics, and quasi-likelihood *F*-tests for ATAC-seq differential accessibility. Gene set enrichment was assessed using Kolmogorov–Smirnov tests with FDR correction. For immunoblotting, band intensity values were normalized to the average intensity of each protein across the blot, adjusted to the corresponding loading control, and expressed as fold change relative to the mean of the respective control (*n* ≥ 3). Data are presented as mean ± SD. For immunofluorescence imaging, twenty representative cells were randomly selected from at least three independent images and quantified using ImageJ. IC_50_ values from the drug screening were derived using GraphPad Prism. Drug interaction analyses were performed using the Zero Interaction Potency (ZIP) model, and absolute synergy scores were summarized across all the tested samples. Statistical methods, cutoffs, and replicate numbers for analyses are provided in the respective figure legends.

### Reporting summary

Further information on research design is available in the Nature Research Reporting Summary linked to this article.

## Supplementary information

Supplementary Material

Uncropped Original Western Blot Files

Original Data - qPCR

Reporting Summary

Differential gene analysis list

Gene set enrichment and string analysis list

## Data Availability

RNA-seq and ATAC-seq data have been deposited in the NCBI GEO database under accession ID: GSE297364 and GSE297366. Mass spectrometry-based proteomics and secretomics data are available via the ProteomeXchange Consortium through the PRIDE repository with dataset identifier PXD041871.
