# Supplementary material for: Epigenetic remodeling via HDAC6 inhibition amplifies anti-tumoral immune responses in myeloid leukemia cells
Source: Cell Death Dis. 2026 Mar 7;17(1):300. doi: 10.1038/s41419-026-08541-3 (PMC13039999; doi:10.1038/s41419-026-08541-3)

Original Data – Uncropped Western Blot Files

Bands highlighted in red:

- were used in the publication
- additional replicates used for the quantification

Figure: 1A

Sample: K562 – wildtype = WT / empty vector control = CTR / HDAC6 KO Clone 1 = C1 / HDAC6 KO Clone 2 = C2

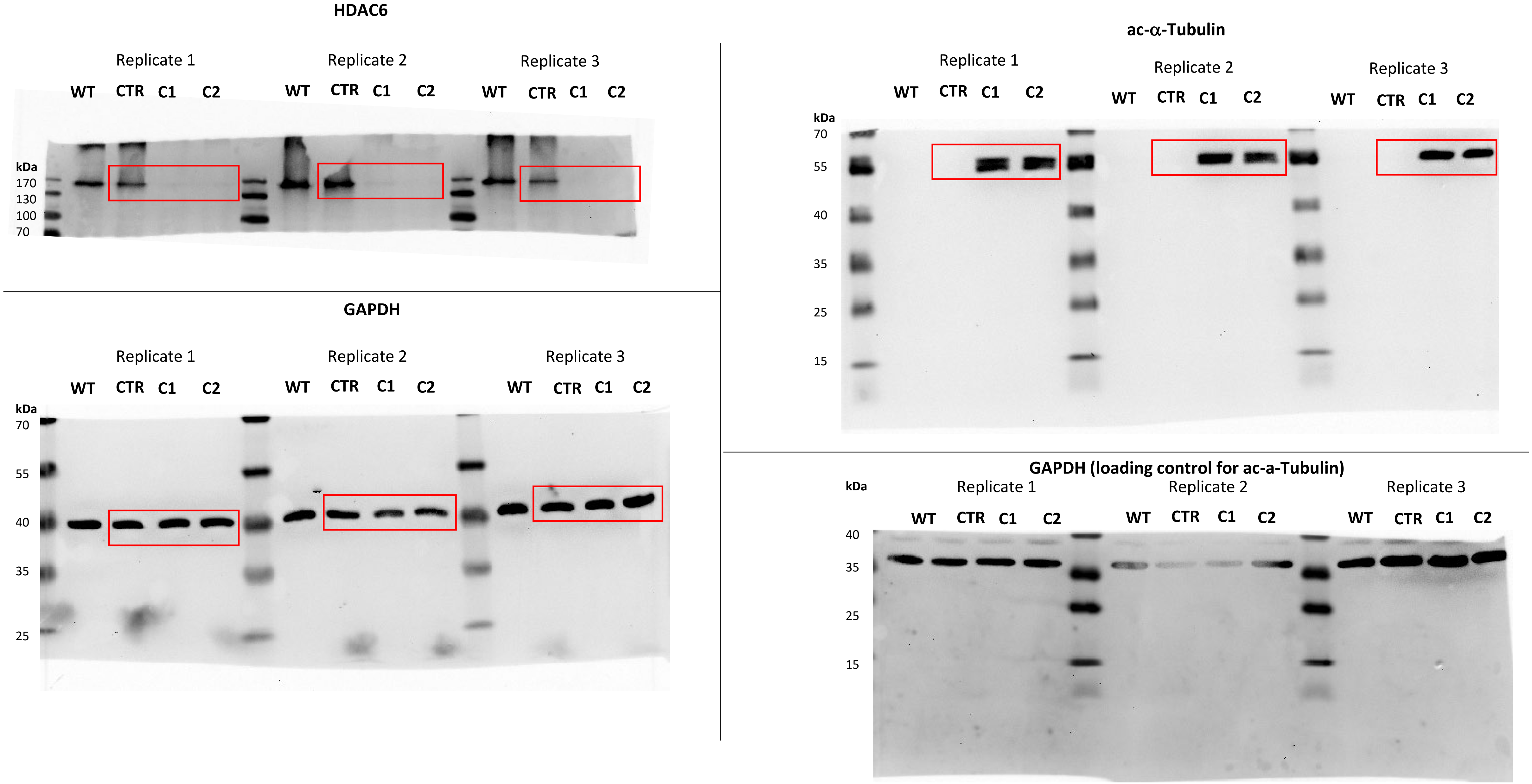

Figure: 1E

Sample: MV4-11 – empty vector control = CTR / HDAC6 KO Clone 1 = C1 / HDAC6 KO Clone 1 = C2

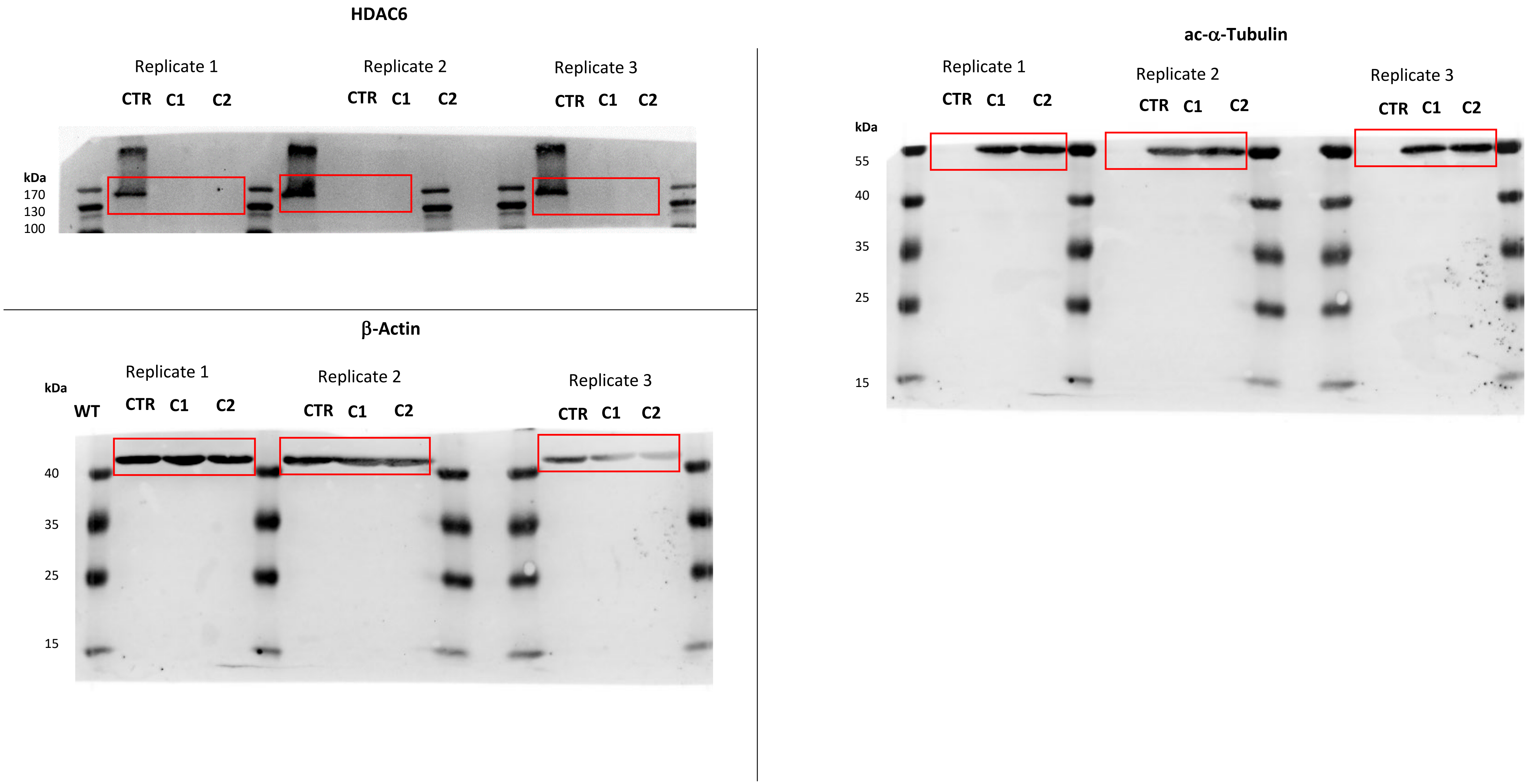

Supplemental Figure: S2E

Sample: KCL22 – wildtype = WT / empty vector control = CTR / HDAC6 KO Clone 1 = C1 / HDAC6 KO Clone 2 = C2

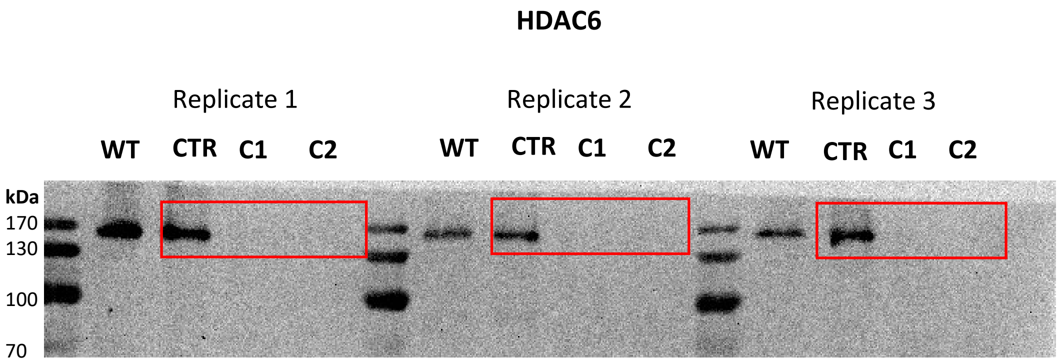

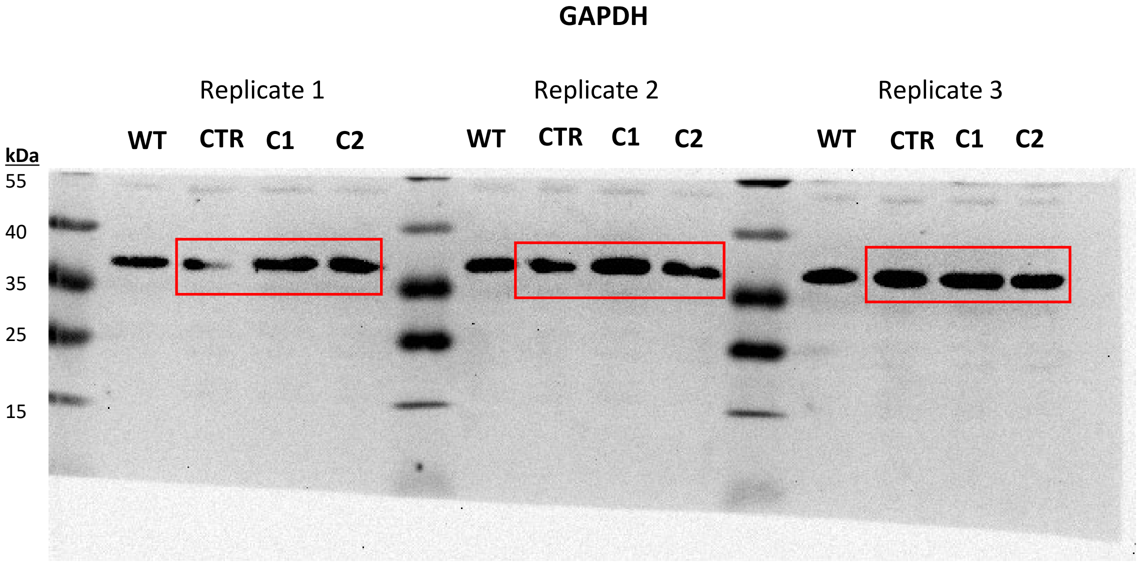

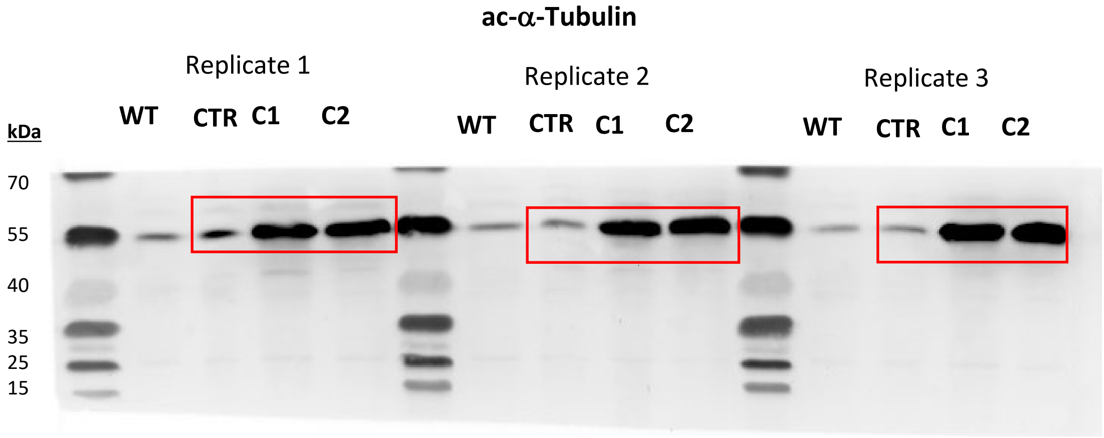

Figure: 2F
Sample: PDX-CML treated with DMSO (D) or 0.5 µM / 1µM / 2µM / 5µM / 10 µM Ricolinostat for 24 hours

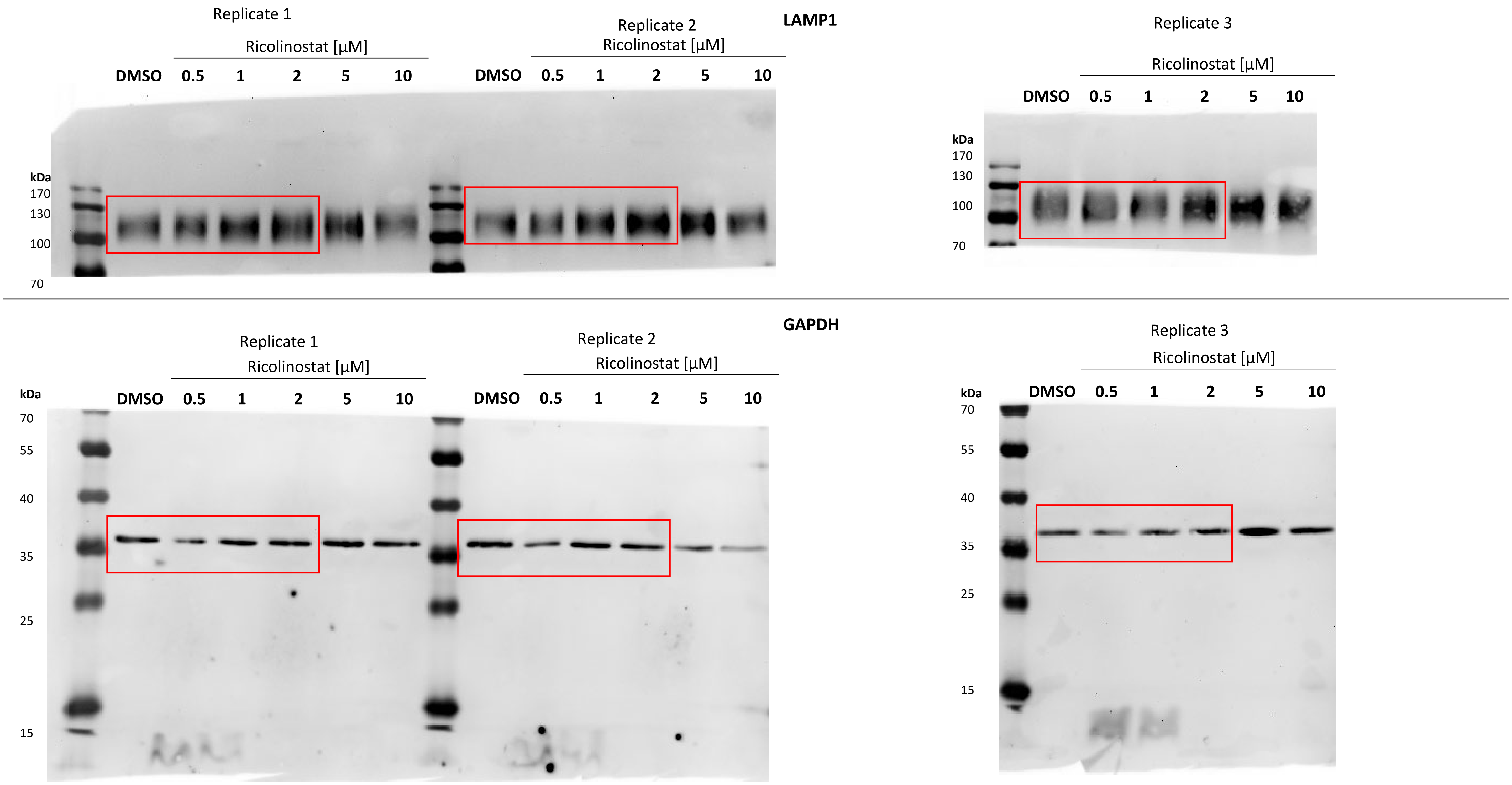

Supplemental Figure: 2G

Sample: MV4-11 – empty vector control = CTR / HDAC6 KO Clone 1 = C1 / HDAC6 KO Clone 2 = C2

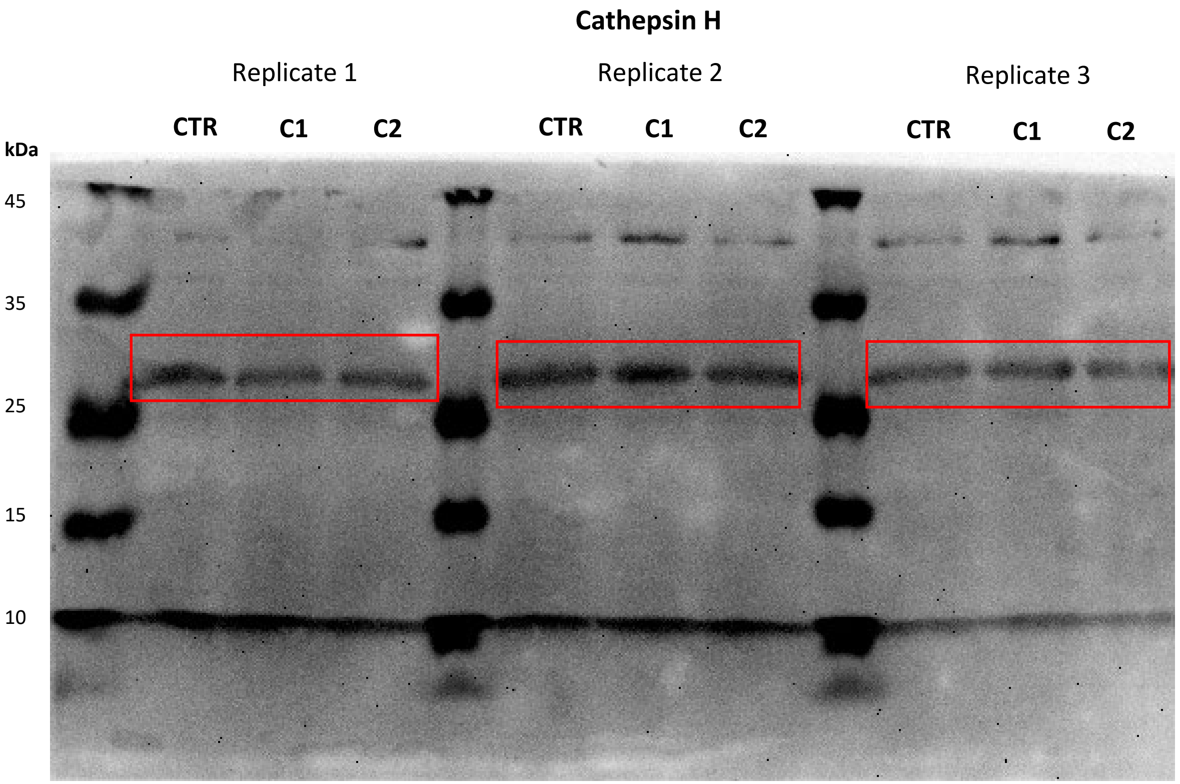

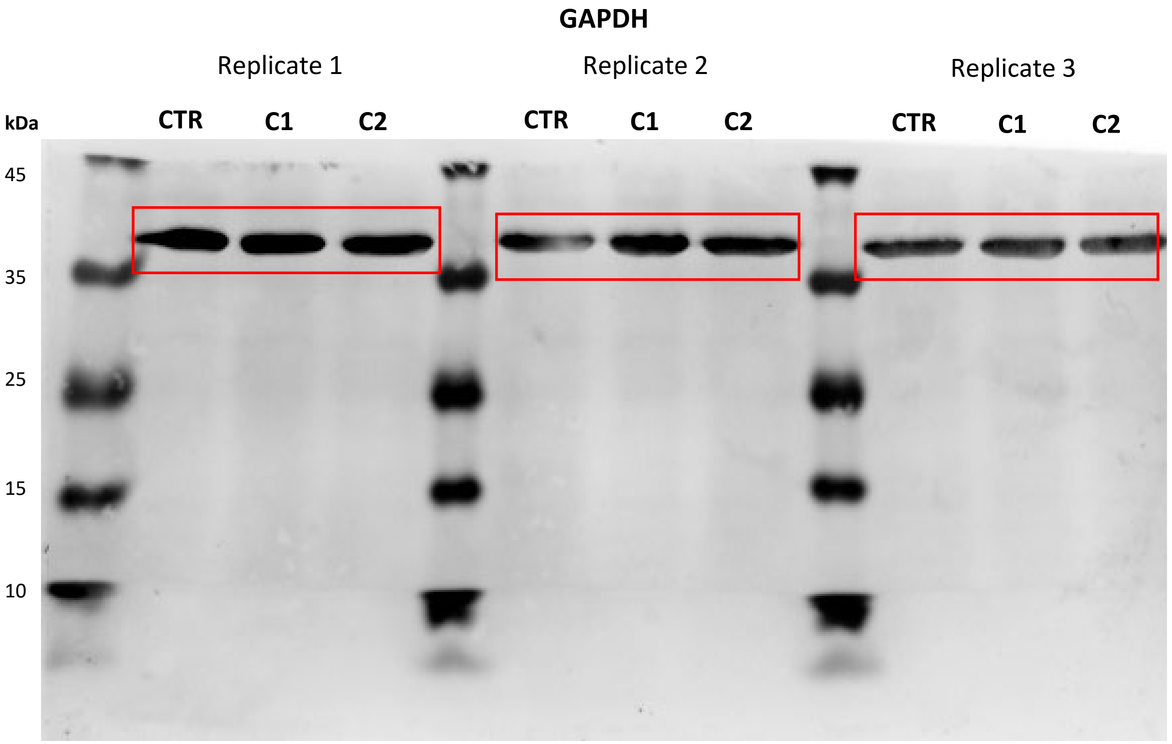

Figure: 4A

Sample: K562 - wildtype = WT / empty vector control = CTR / HDAC6 KO Clone 1 = C1 / HDAC6 KO Clone 2 = C2

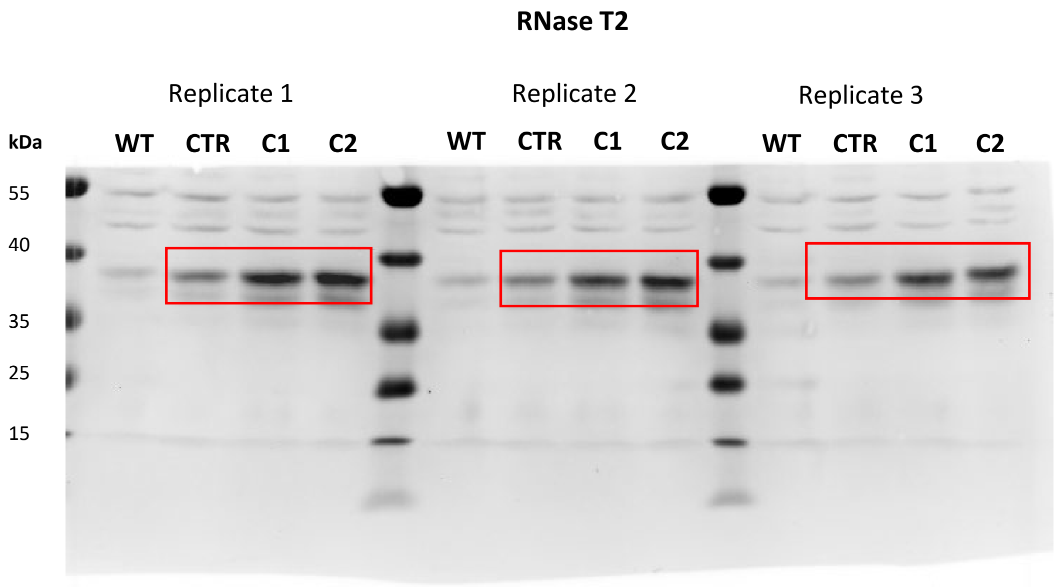

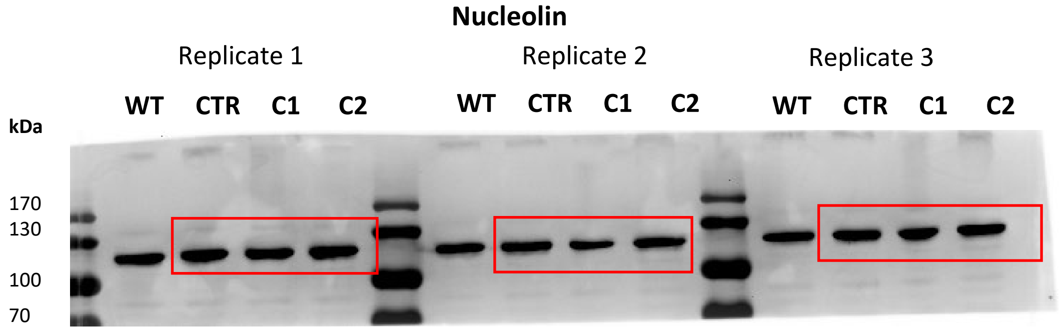

Supplemental Figure: S4A

Sample: K562 supernatant – empty vector control = CTR / HDAC6 KO Clone 1 = C1 / HDAC6 KO Clone 2 = C2

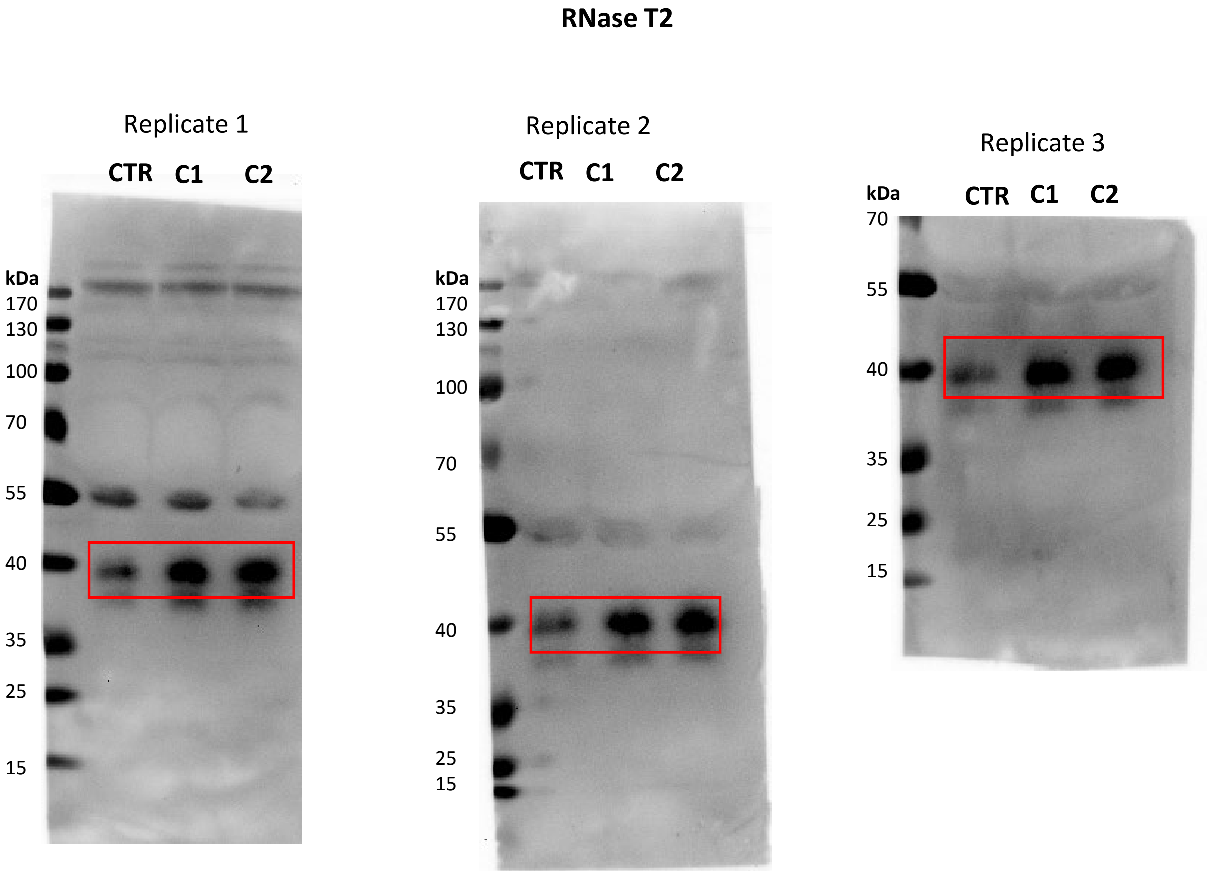

Supplemental Figure: 4B

Sample: K562 wildtype = WT / HDAC6 KO Clone 2 = C2 / HDAC6 KO Clone 2 with KI =C2/KI

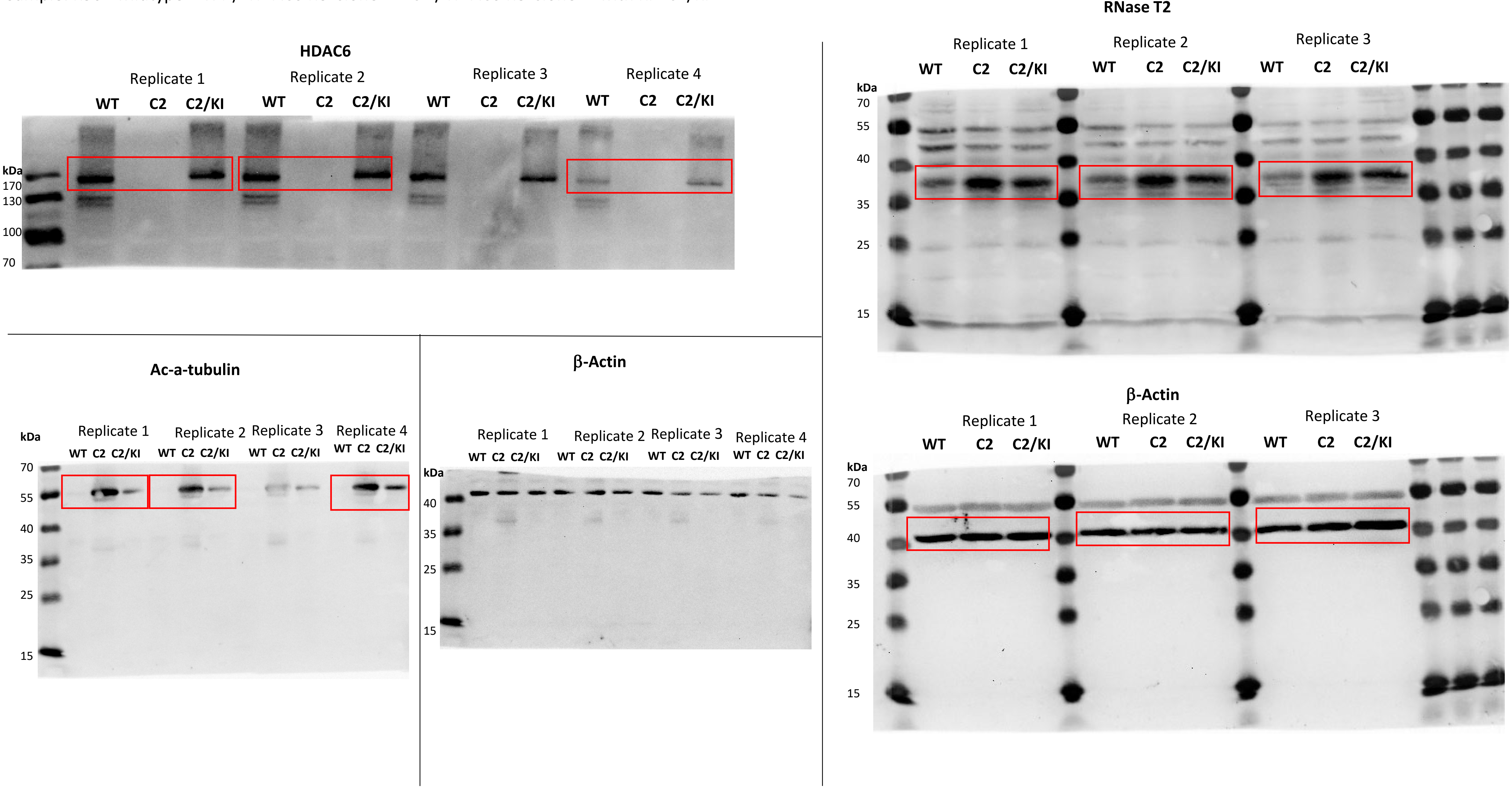

Figure: 4B

Sample: KCL22 - wildtype = WT / empty vector control = CTR / HDAC6 KO Clone 1 = C1 / HDAC6 KO Clone 2 = C2

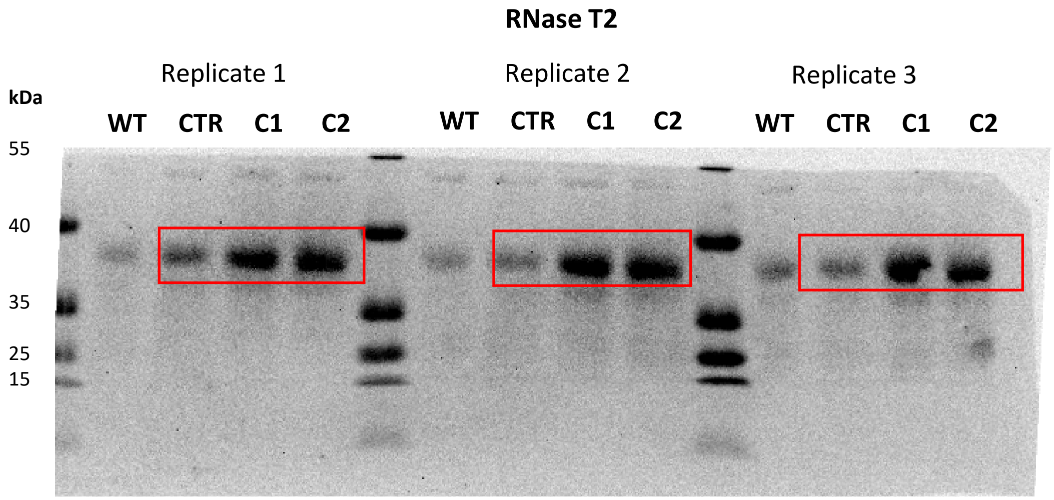

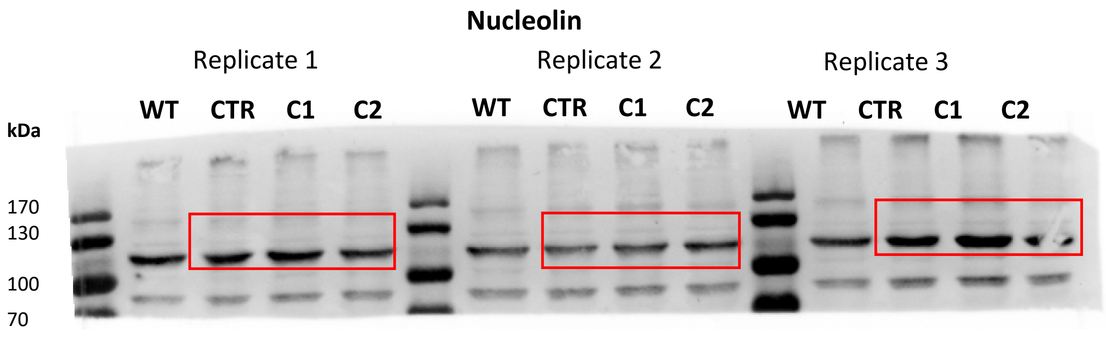

Figure: 4C

Sample: MV4-11 - empty vector control = CTR / HDAC6 KO Clone 1 = C1 / HDAC6 KO Clone 2 = C2

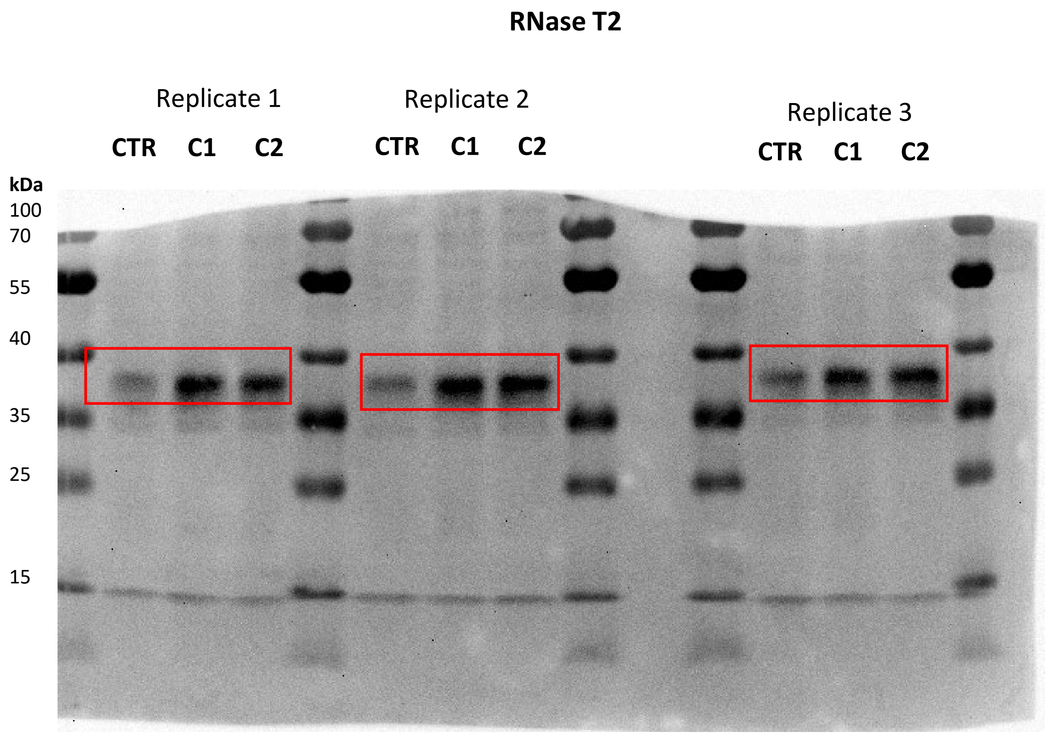

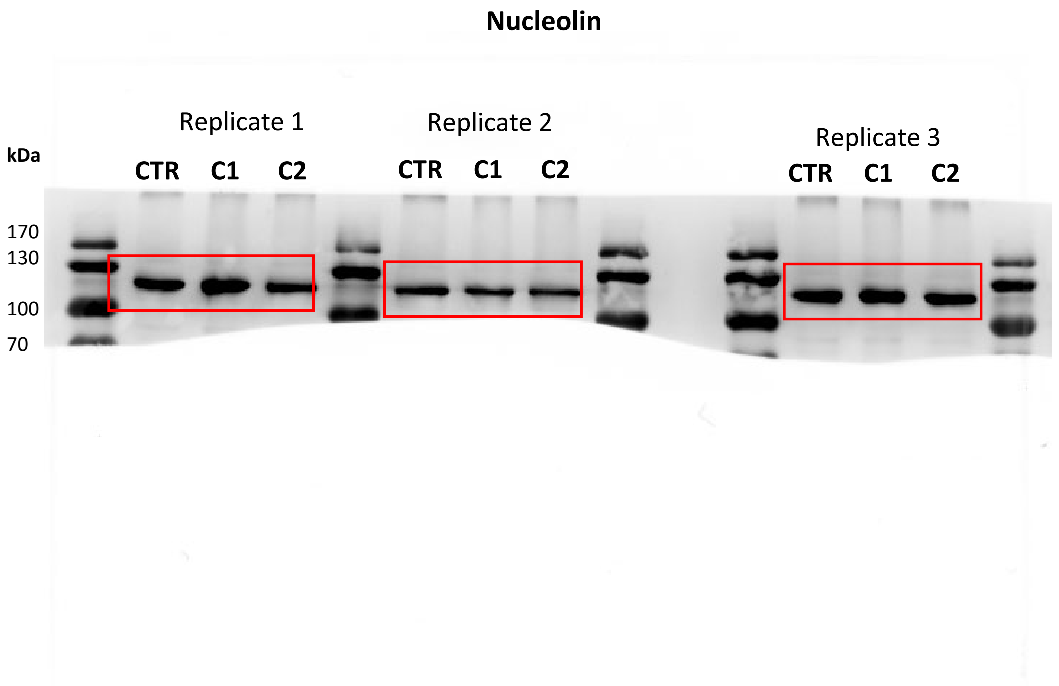

Supplemental Figure: S4C

Sample: NALM20 – wildtype = WT / empty vector control = CTR / HDAC6 KD shRNA 1 = sh1 / HDAC6 KD shRNA 2 = sh2

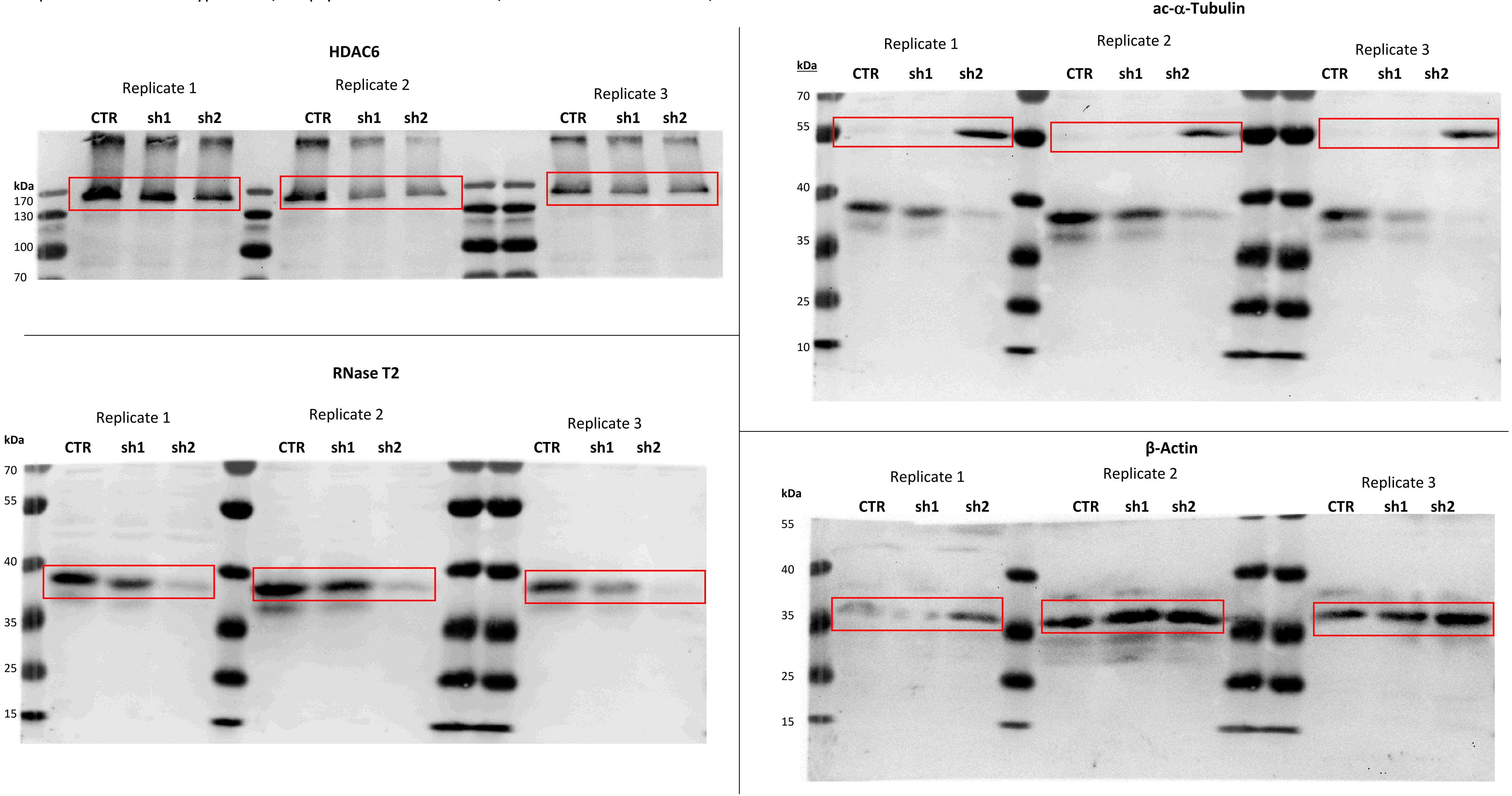

Supplemental Figure: S4D

Sample: PDX-B-ALL2 –empty vector control = CTR / HDAC6 KD shRNA 1 = sh1 / HDAC6 KD shRNA 2 = sh2

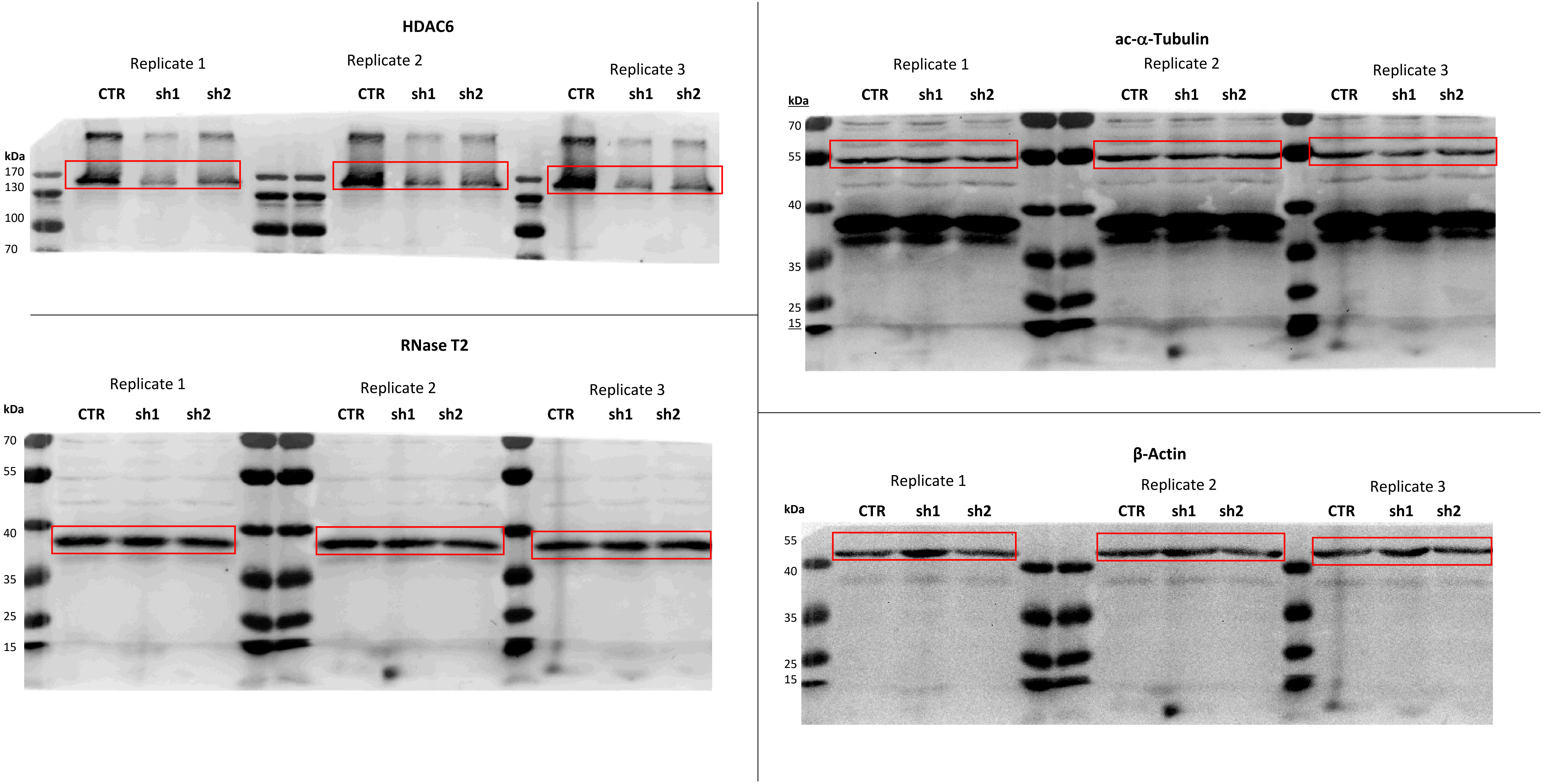

Supplemental Figure: S4E
Sample: NALM6 – wildtype = WT / empty vector control = CTR / HDAC6 KO Clone 1 = C1 / HDAC6 KO Clone 2 = C2

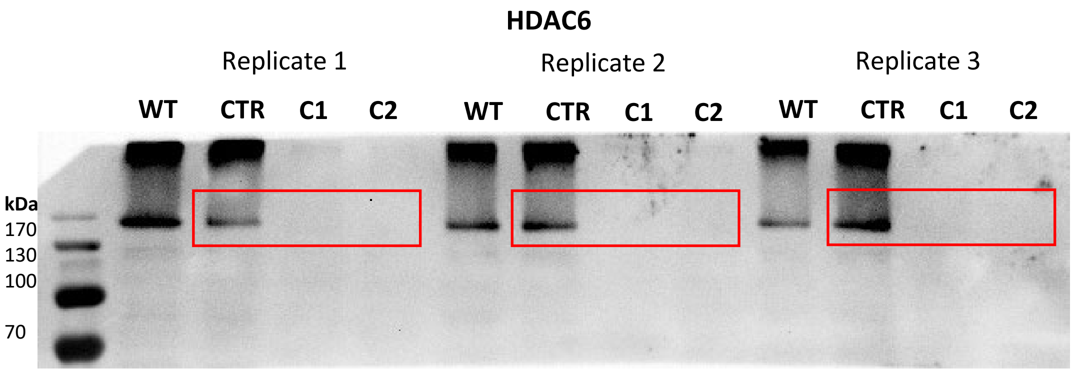

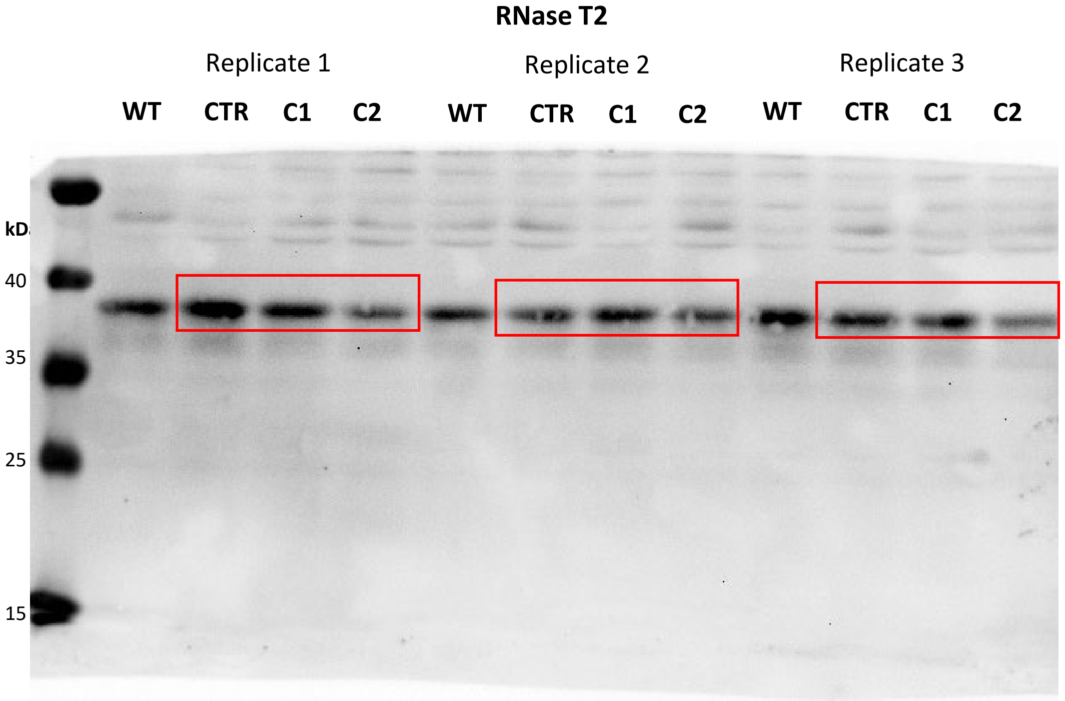

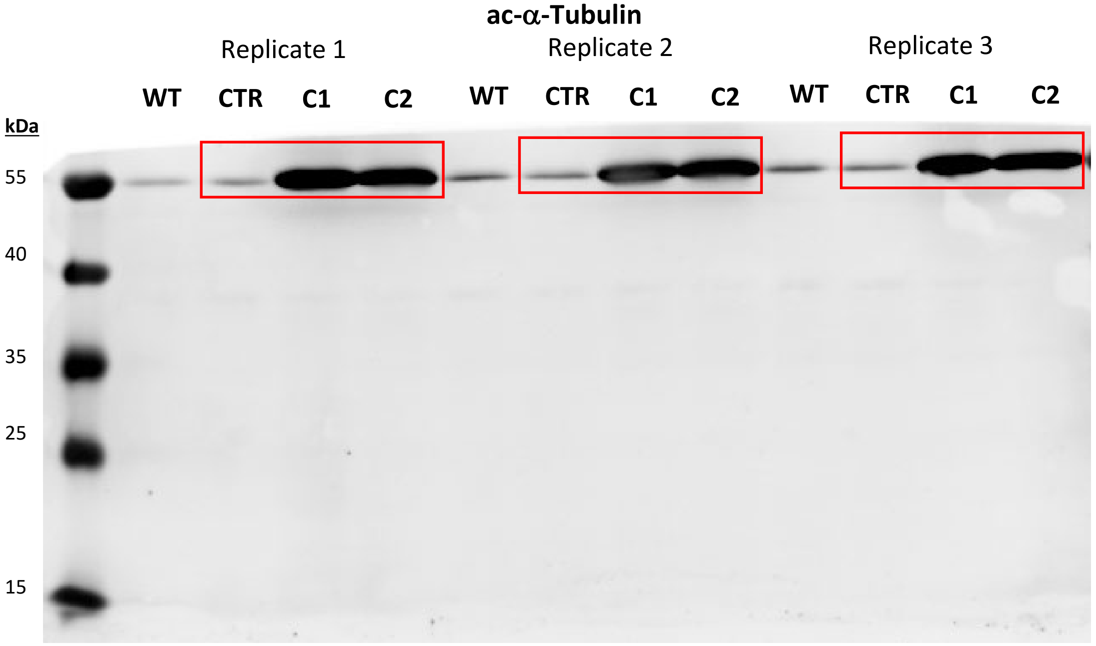

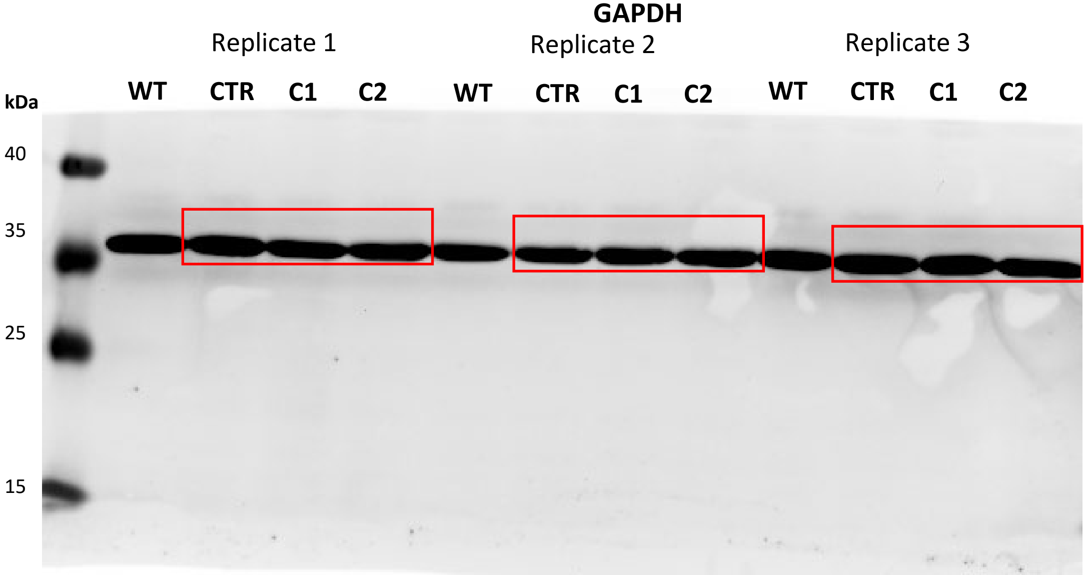

Supplemental Figure: S4F

Sample: NALM6 – wildtype = WT / empty vector control = CTR / HDAC6 KD shRNA 1 = sh1 / HDAC6 KD shRNA 2 = sh2

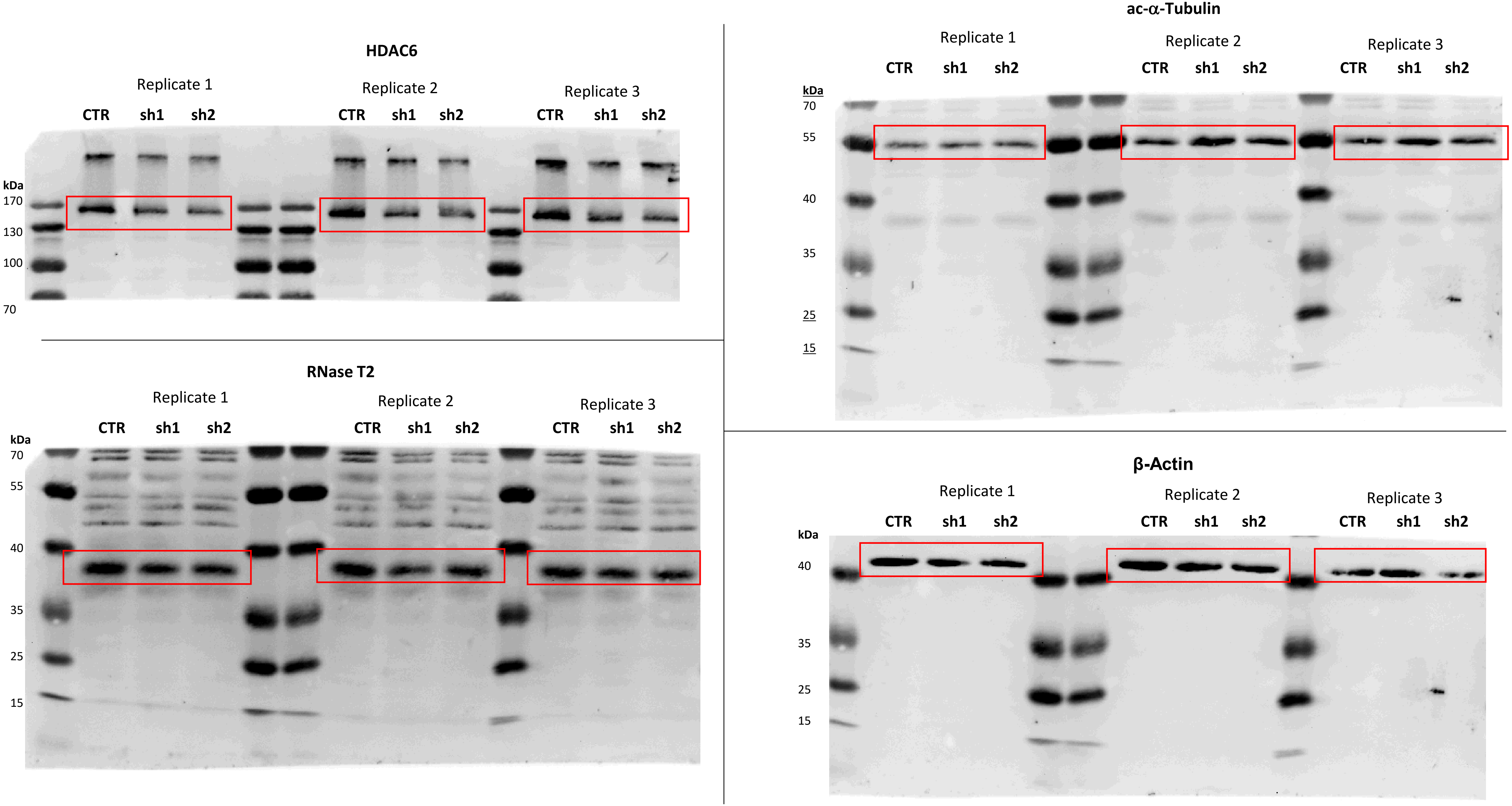

Figure: 4D

Sample: K562 treated with DMSO (D) or 5µM / 10 µM Ricolinostat for 24 hours / 48 hours

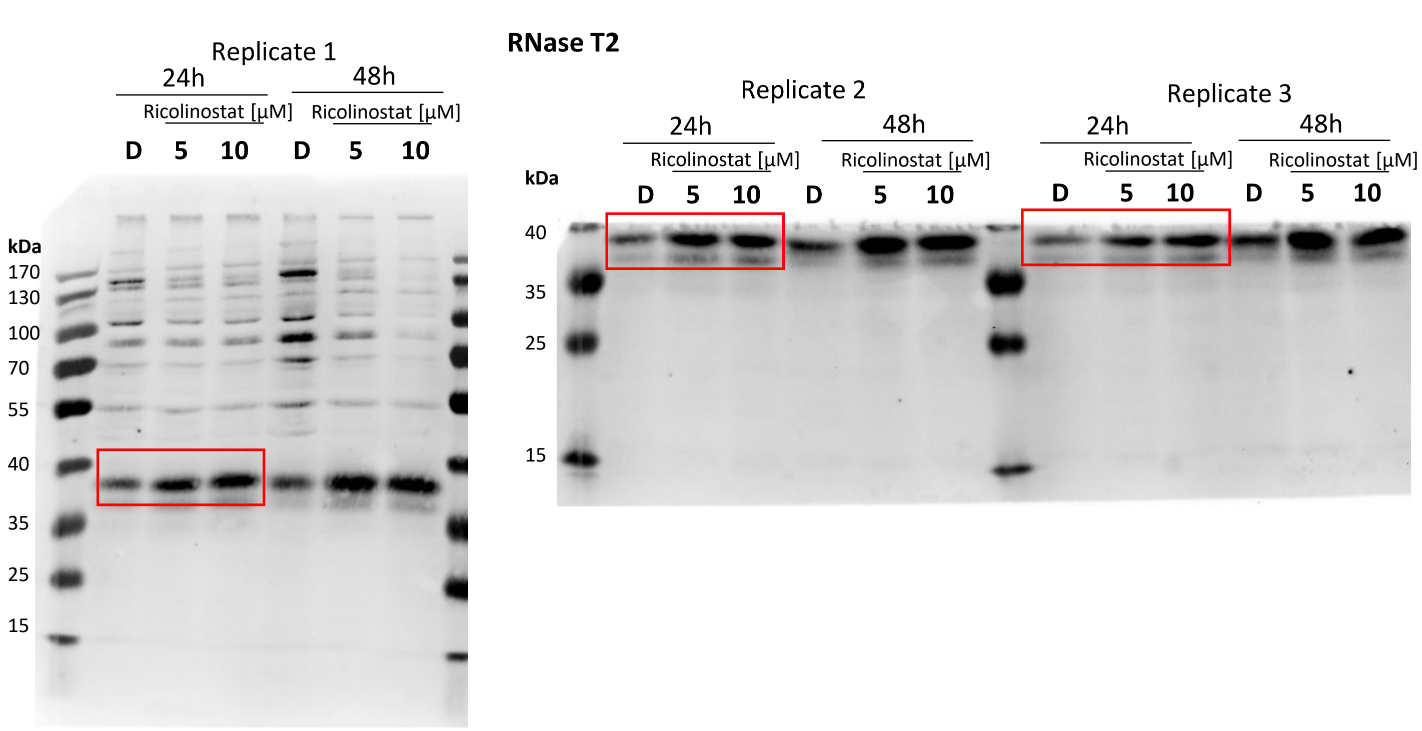

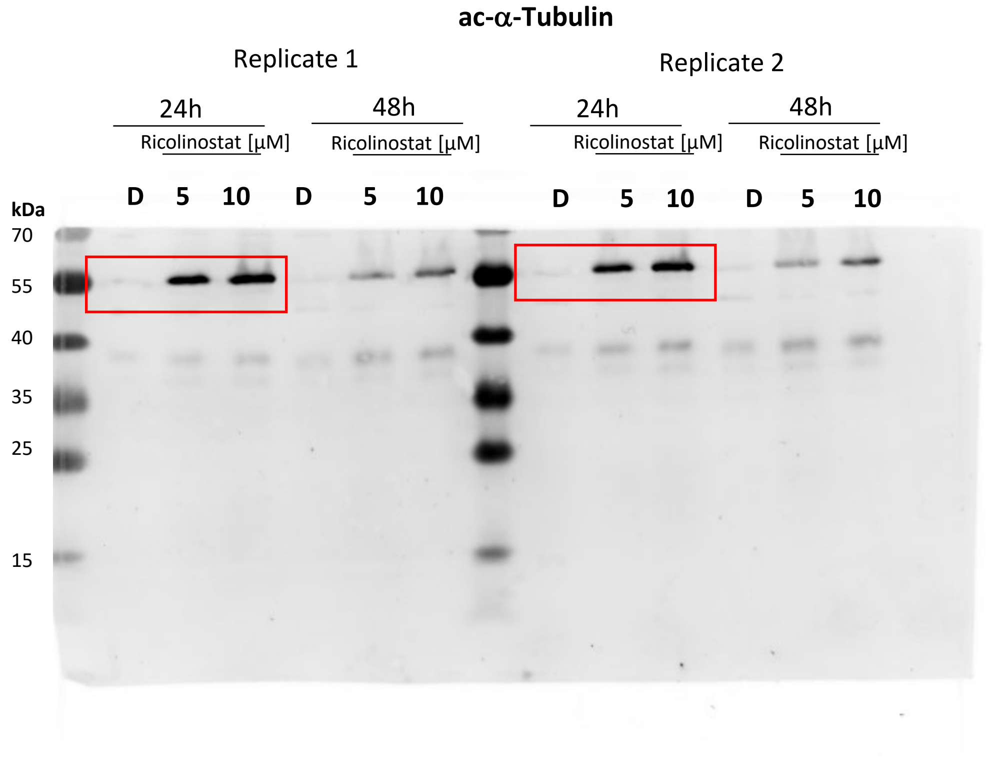

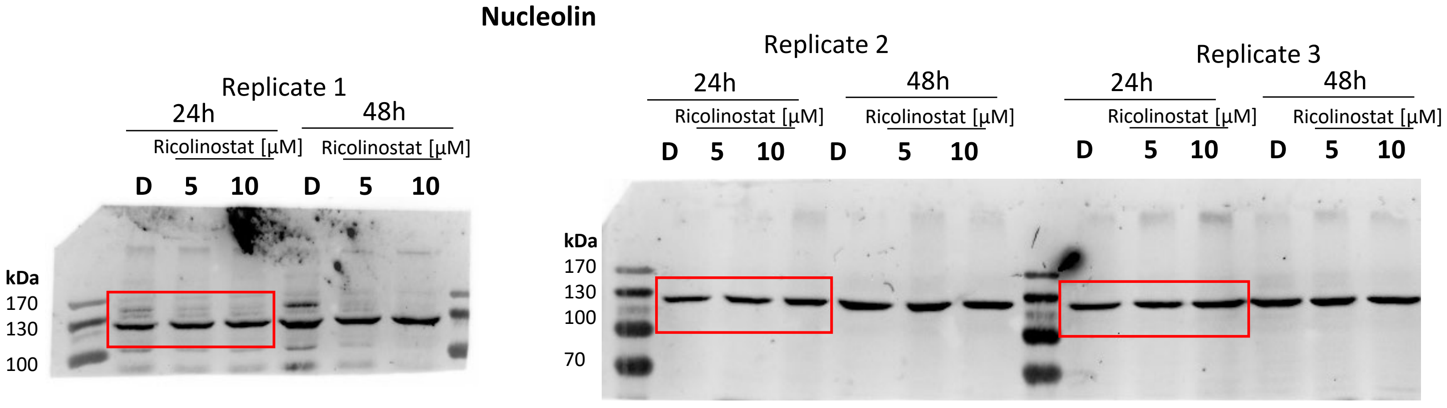

Figure: 4E

Sample: THP1 treated with DMSO (D) or 1 µM / 2 µM Ricolinostat for 24 hours / 48 hours

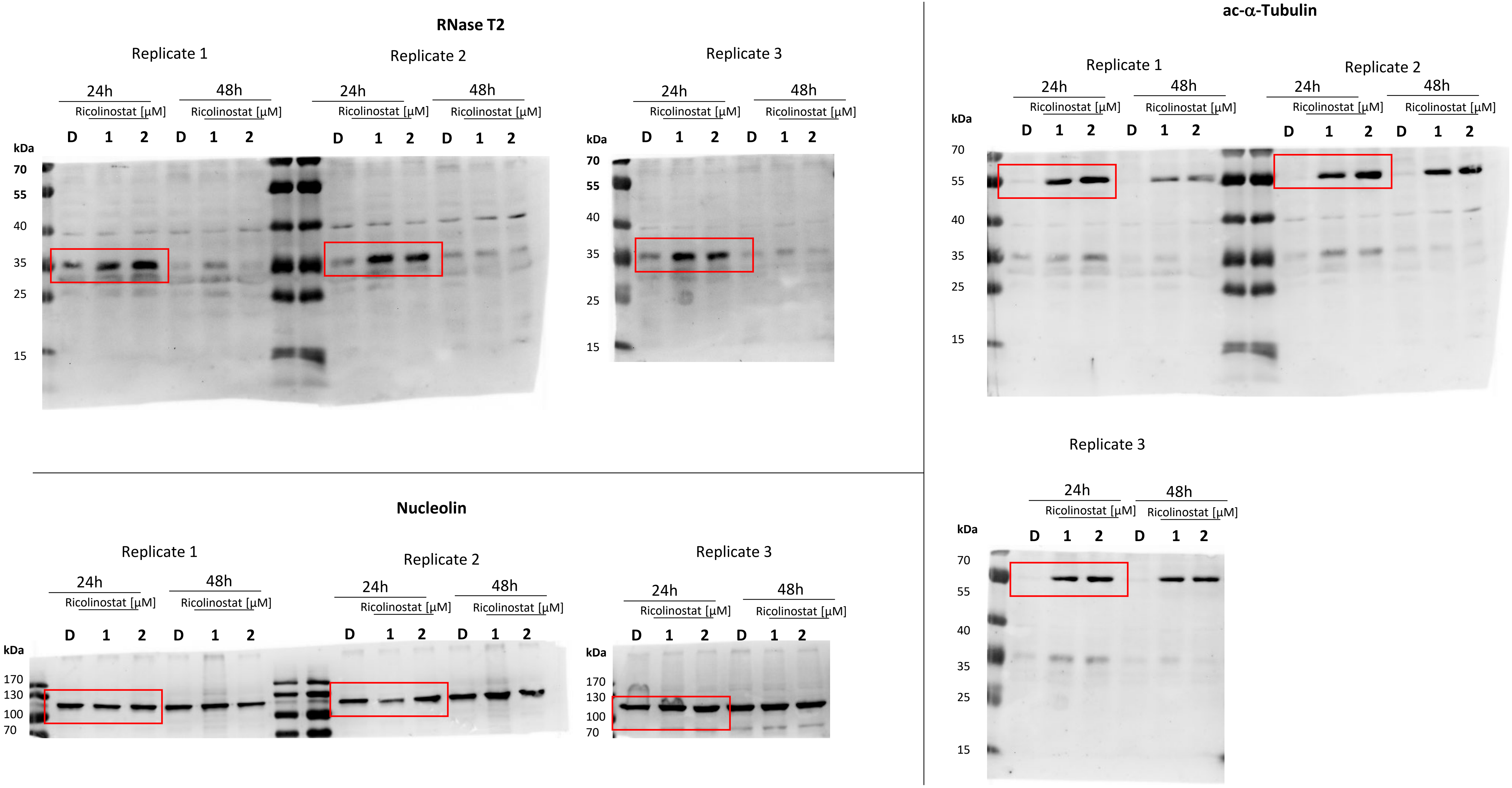

Figure: 4F

Sample: HL60 treated with DMSO (D) or 1 µM / 2 µM Ricolinostat for 24 hours / 48 hours

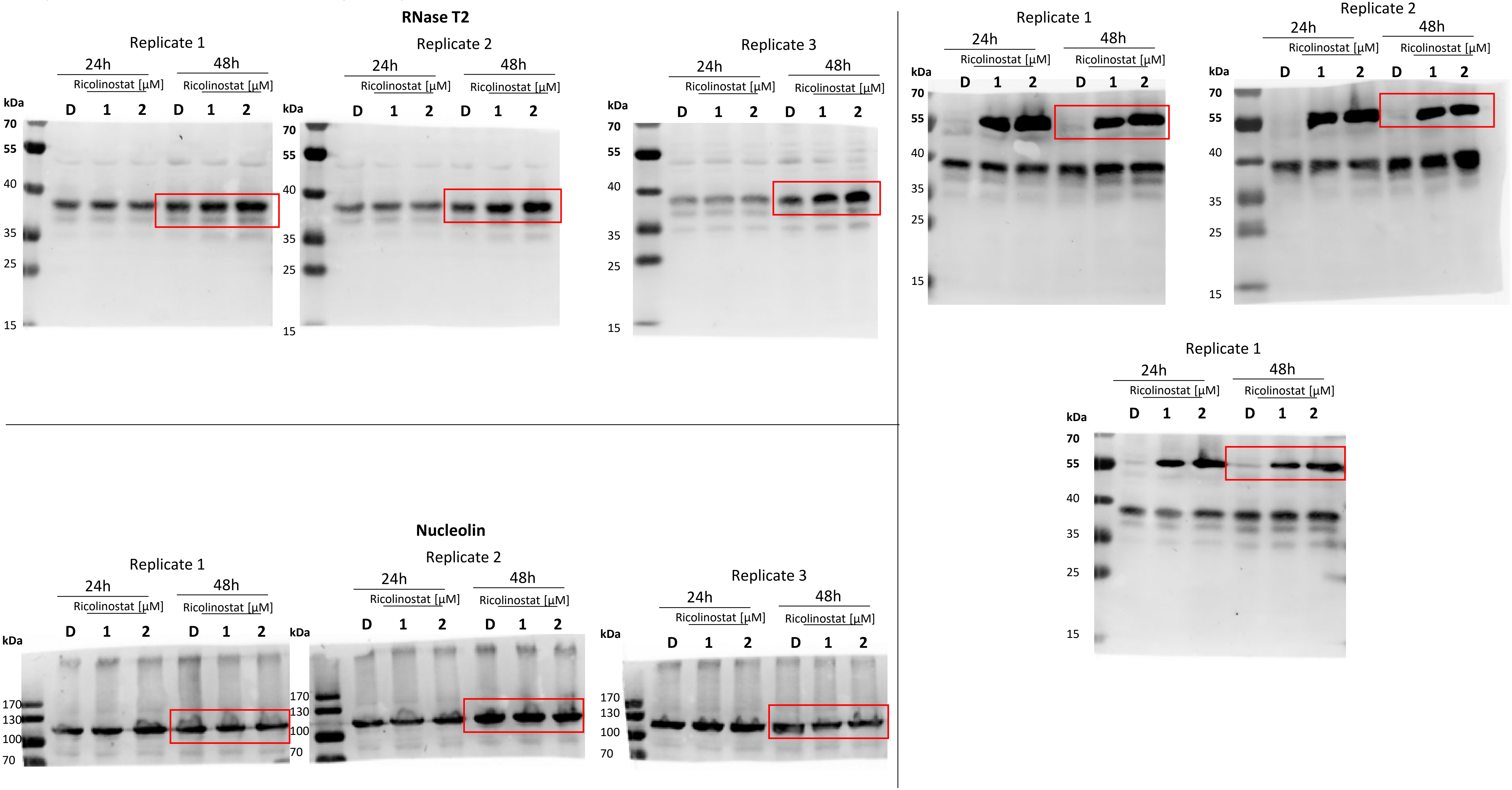

Figure: 4G

Sample: PDX-CML treated with DMSO (D) or 0.5 µM / 1µM / 2µM / 5µM / 10 µM Ricolinostat for 24 hours

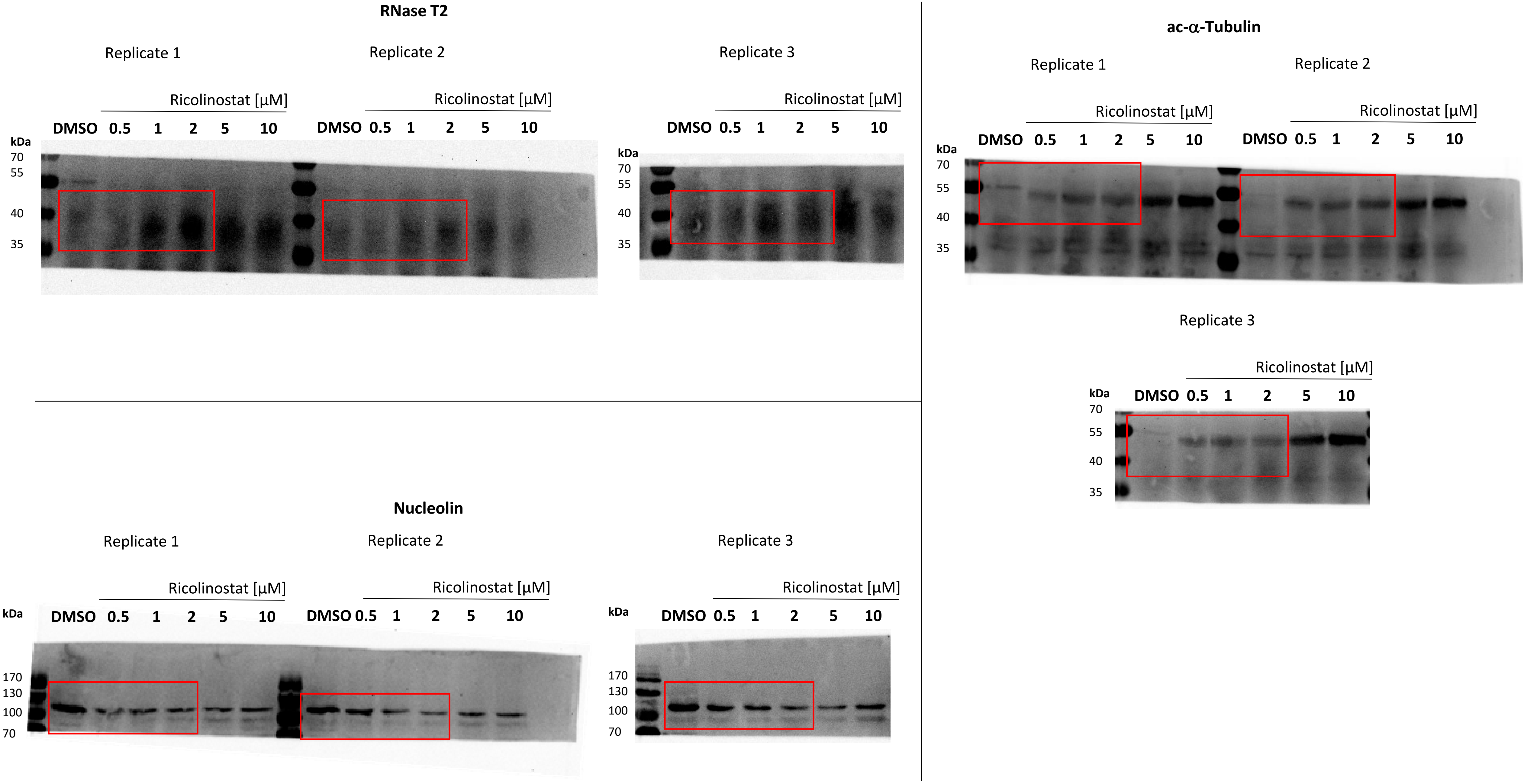

Figure: 4H

Sample: PDX-AML treated with DMSO (D) or 1µM / 2µM / 5µM Ricolinostat for 24 hours

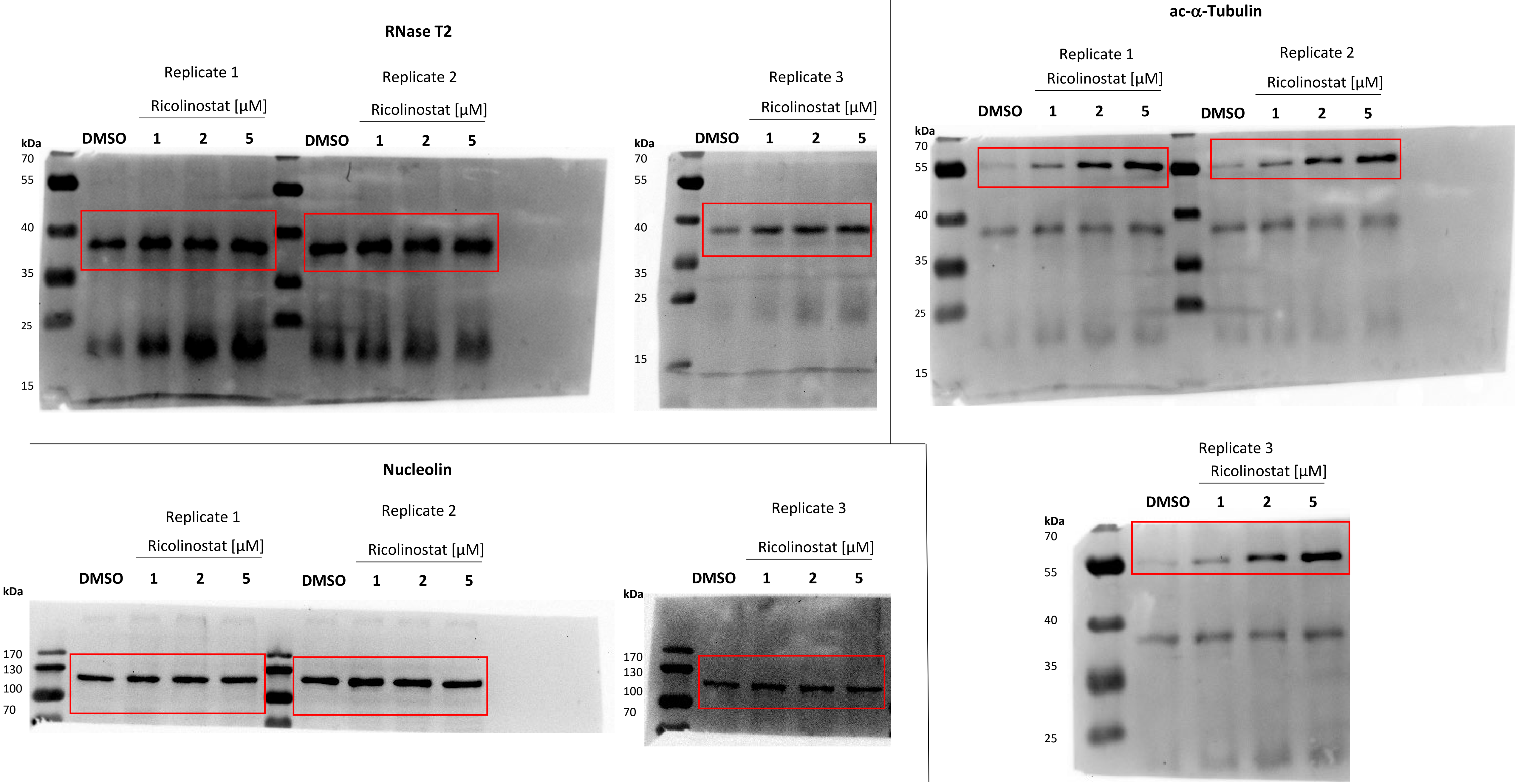

Supplemental Figure: S5F

Sample: C1498 treated with with DMSO (D) or 0.25µM / 0.5 µM Ricolinostat for 24 hours

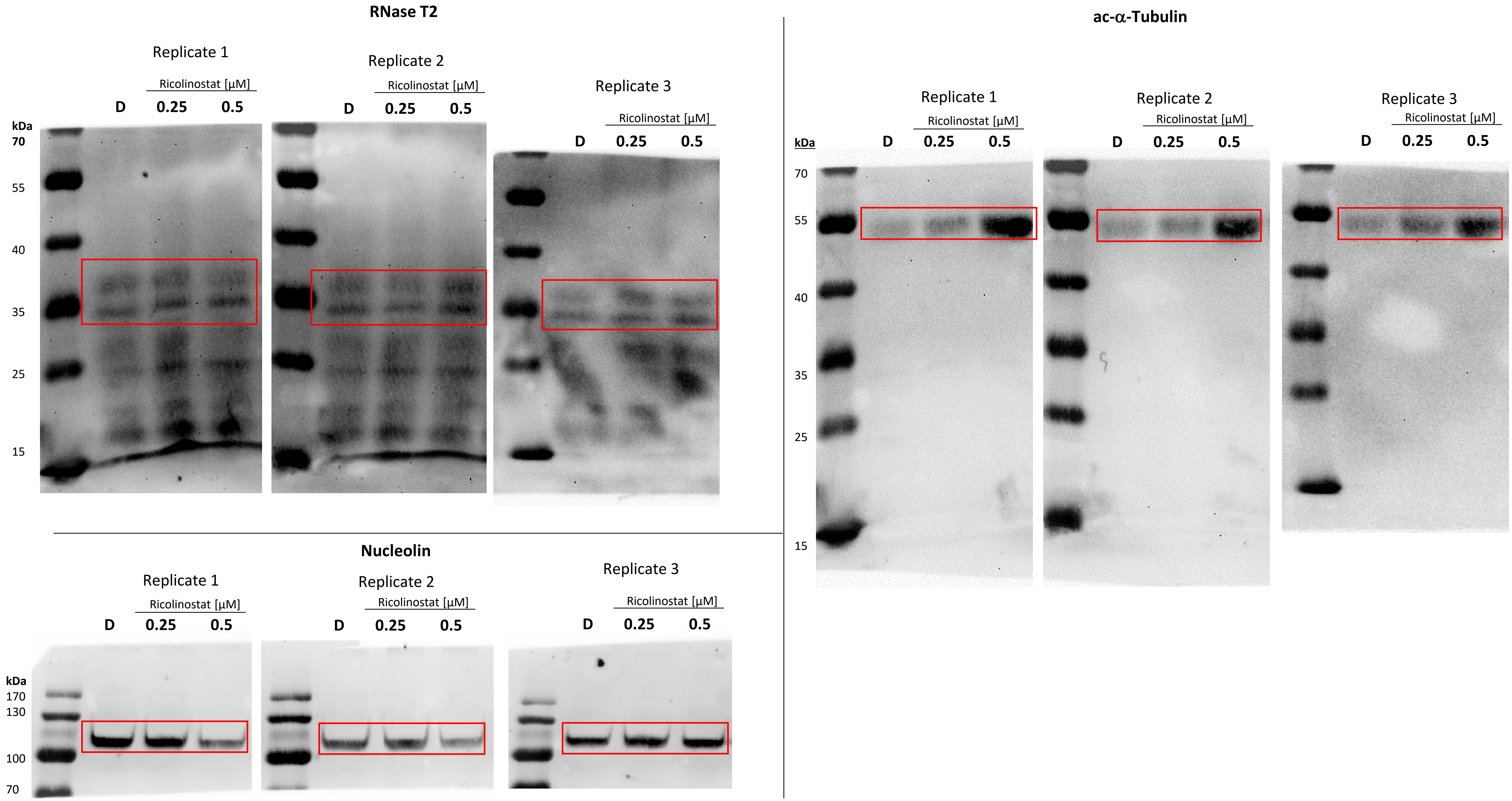

Supplemental Figure: S5G

Sample: C1498 – empty vector control = CTR / HDAC6 KO Clone 1 = C1 / HDAC6 KO Clone 2 = C2

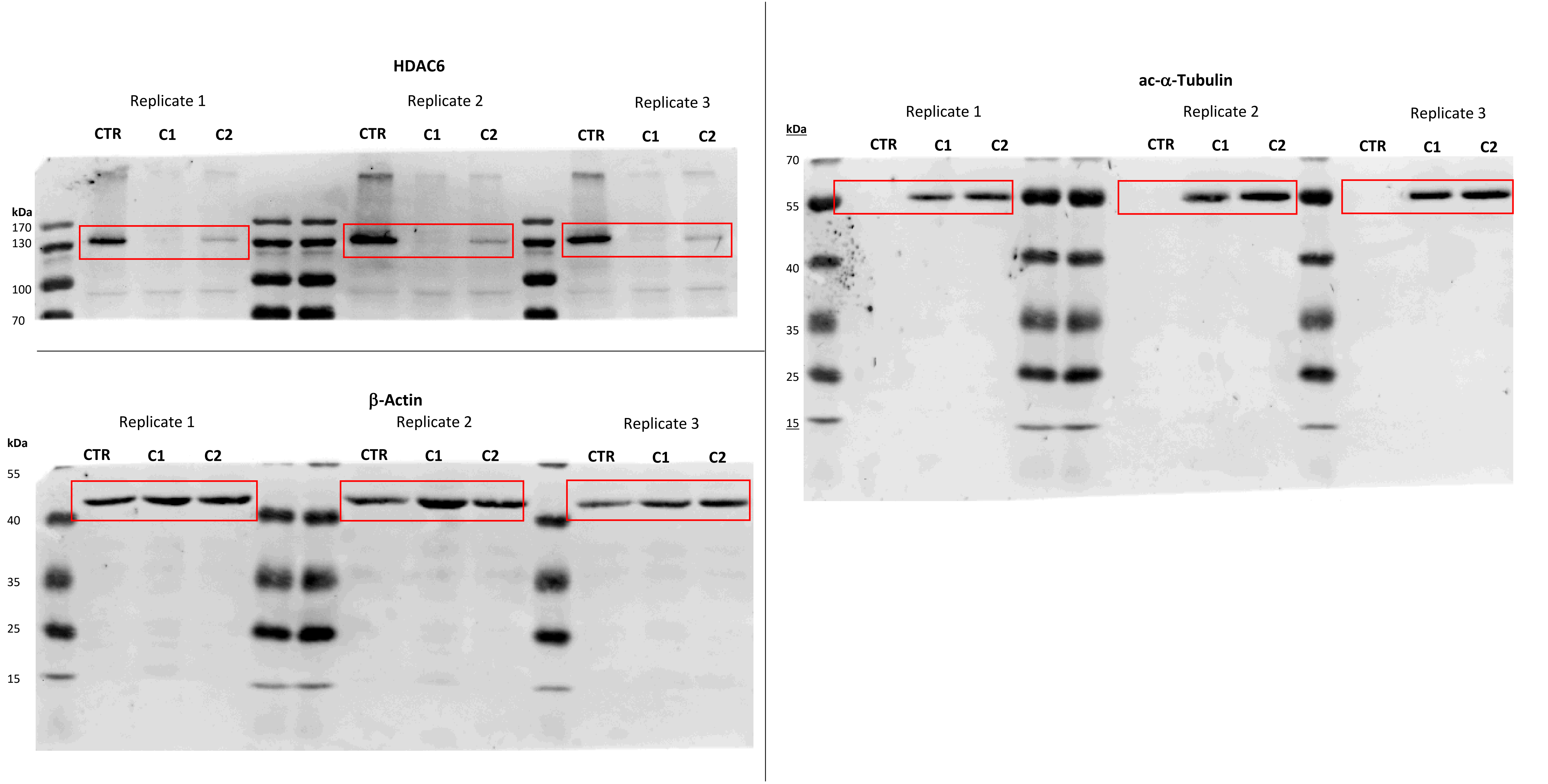

Figure: 6E

Sample: K562 – empty vector control = CTR / HDAC6 KO Clone 2 = C2 treated with DMSO = D / 1µM Clofarabine =Cy / 2 µM Cytarabine =Cl

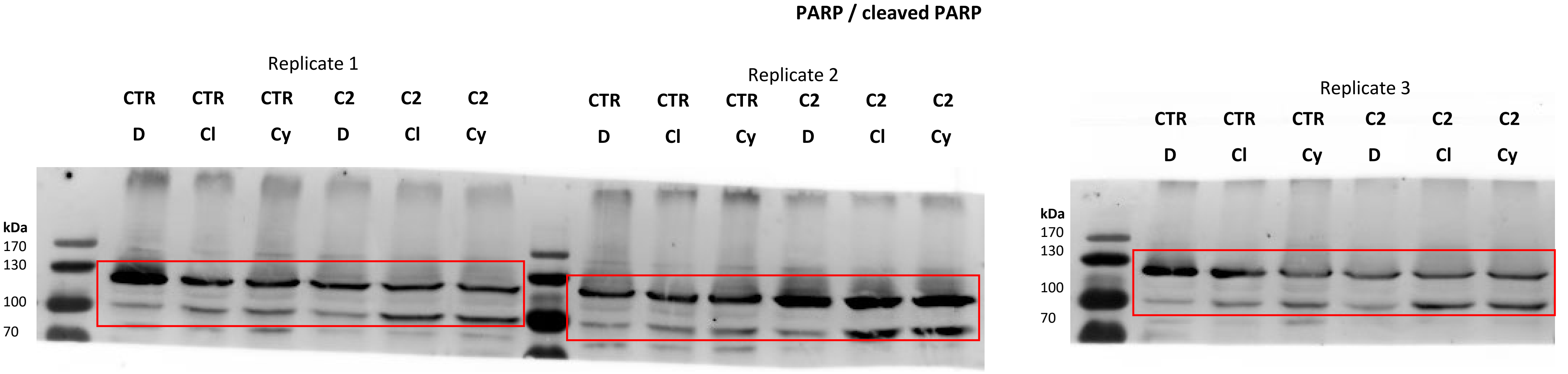

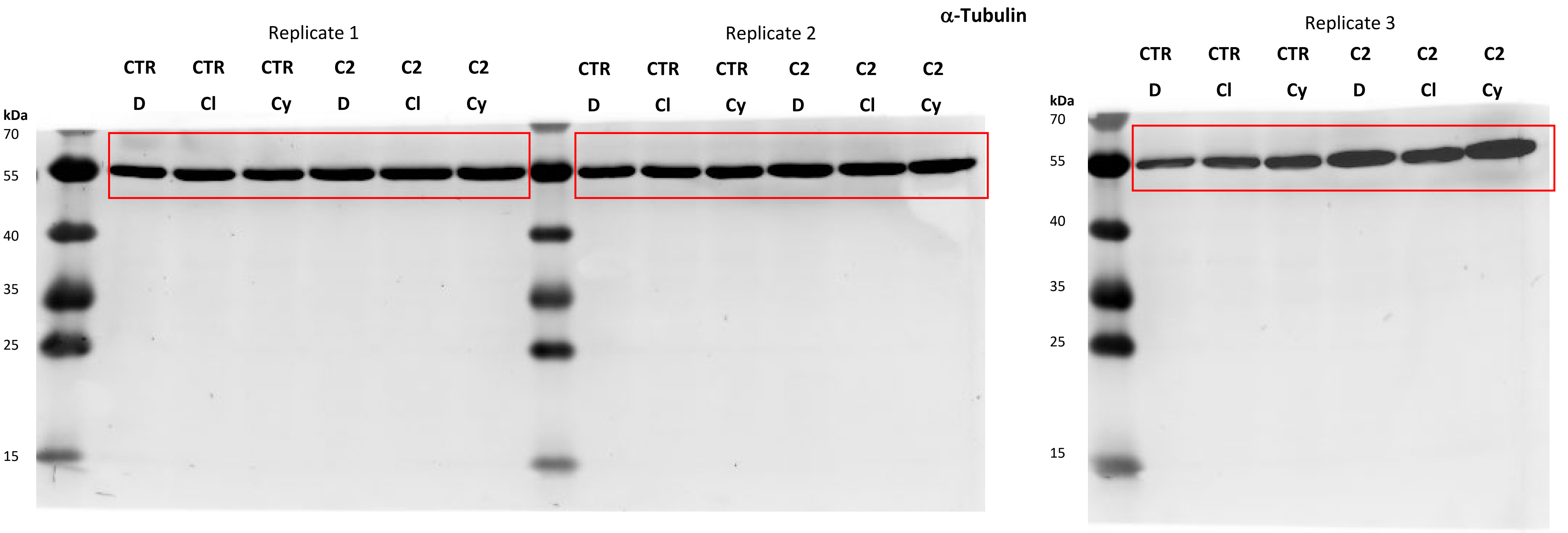

Supplemental Figure: 6G

Sample: K562 – empty vector control = CTR / HDAC6 KO Clone 1 = C1 / HDAC6 KO Clone 2 = C2

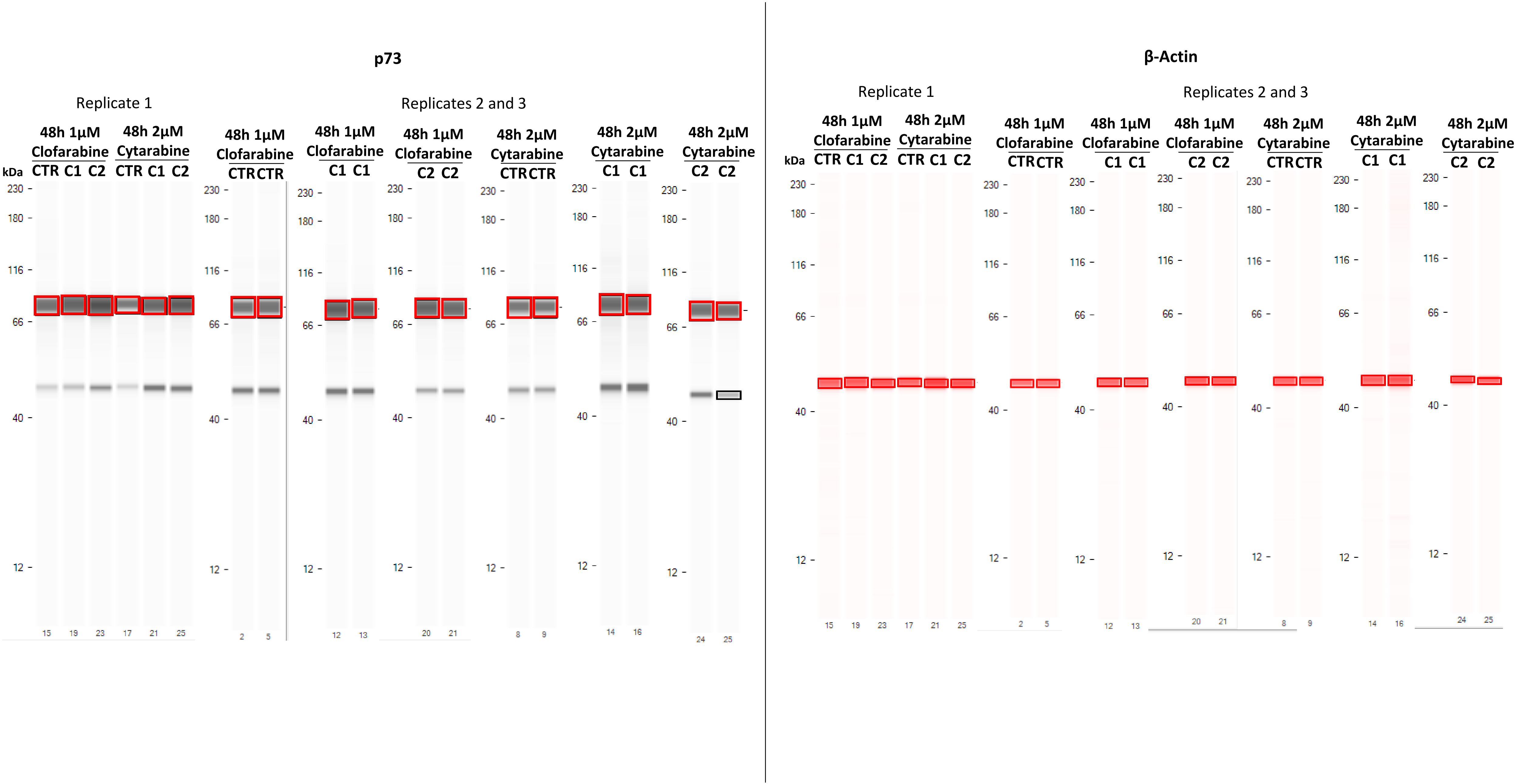

Supplemental Figure: 6H

Sample: MV4-11 – empty vector control = CTR / HDAC6 KO Clone 1 = C1 / HDAC6 KO Clone 2 = C2

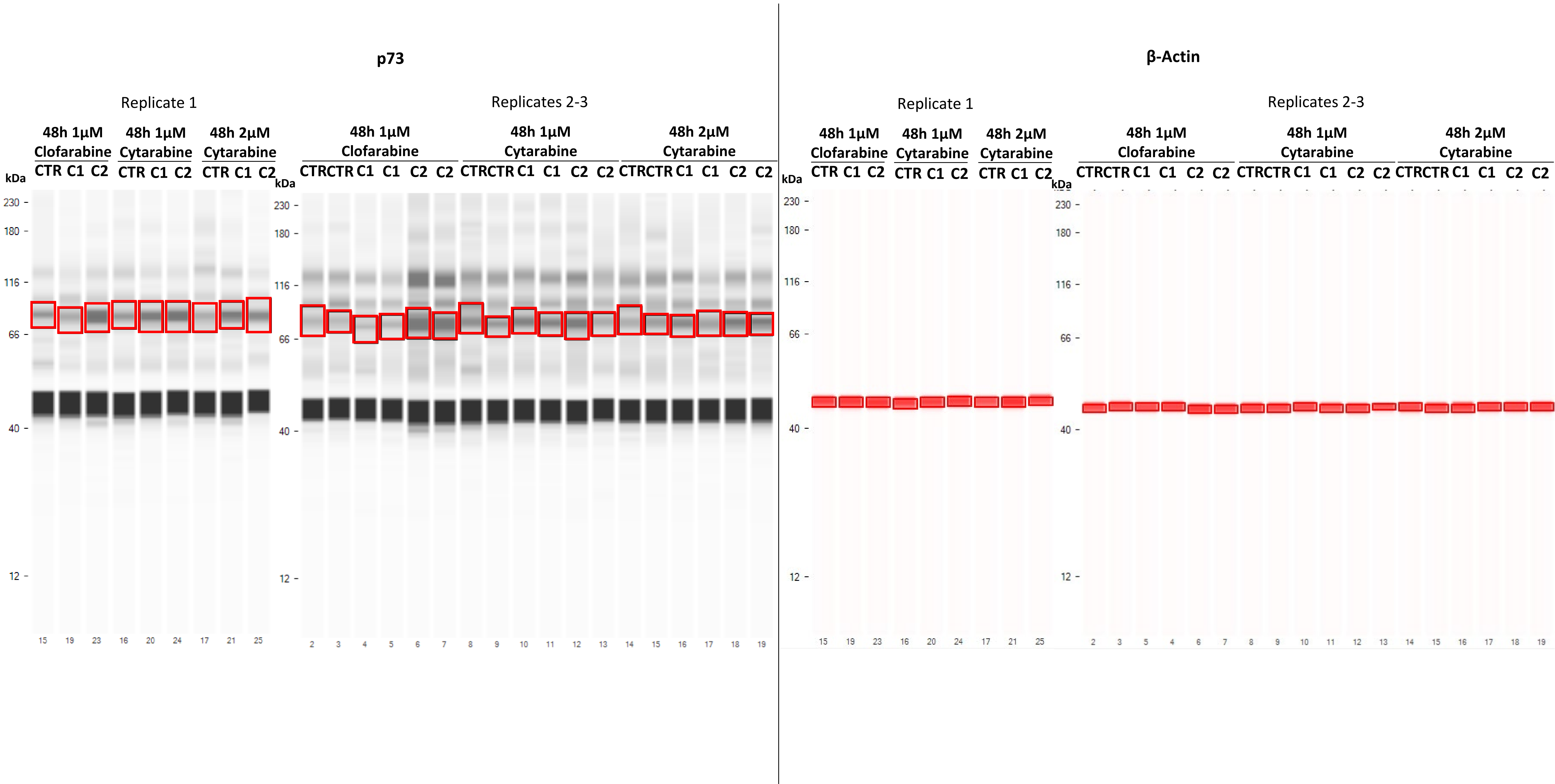

Supplement: Supplementary file 2 — Uncropped Original Western Blot Files [file 41419_2026_8541_MOESM2_ESM.pdf]
