# Supplementary material for: Epigenetic remodeling via HDAC6 inhibition amplifies anti-tumoral immune responses in myeloid leukemia cells
Source: Cell Death Dis. 2026 Mar 7;17(1):300. doi: 10.1038/s41419-026-08541-3 (PMC13039999; doi:10.1038/s41419-026-08541-3)
Supplement: Supplementary file 3 — Original Data - qPCR [file 41419_2026_8541_MOESM3_ESM.pdf]

Original Data- qPCR

Well	Sample	Sample type	Condition	Target	Dye	Cq	Melt Temperature	Peak Height	Begin Temperature	End Temperature
A01	K562	Cell line	DMSO treated	RNase T2	SYBR green	24,11	79,50	1492,09	74,50	91,00
A02	K562	Cell line	DMSO treated	RNase T2	SYBR green	23,79	80,00	1796,64	74,50	93,00
A03	K562	Cell line	DMSO treated	RNase T2	SYBR green	23,83	80,00	1755,02	75,00	93,50
A10	K562	Cell line	5 µM Ricolinostat treated	RNase T2	SYBR green	22,89	80,00	1618,75	75,00	93,50
A11	K562	Cell line	5 µM Ricolinostat treated	RNase T2	SYBR green	23,25	80,00	1526,15	75,00	95,00
A12	K562	Cell line	5 µM Ricolinostat treated	RNase T2	SYBR green	23,20	80,00	1658,02	75,00	92,50
A13	K562	Cell line	10 µM Ricolinostat treated	RNase T2	SYBR green	22,37	80,00	1588,62	75,00	94,00
A14	K562	Cell line	10 µM Ricolinostat treated	RNase T2	SYBR green	22,98	80,00	1699,39	75,00	93,00
A15	K562	Cell line	10 µM Ricolinostat treated	RNase T2	SYBR green	23,72	80,00	1638,73	75,00	91,50
B01	K562	Cell line	DMSO treated	B2M	SYBR green	18,49	81,50	2696,47	77,00	94,00
B02	K562	Cell line	DMSO treated	B2M	SYBR green	18,50	81,50	2778,59	77,00	93,50
B03	K562	Cell line	DMSO treated	B2M	SYBR green	18,74	81,50	2650,85	77,00	92,50
B10	K562	Cell line	5 µM Ricolinostat treated	B2M	SYBR green	18,47	81,50	2602,31	77,00	94,00
B11	K562	Cell line	5 µM Ricolinostat treated	B2M	SYBR green	18,60	81,50	2490,75	77,00	93,00
B12	K562	Cell line	5 µM Ricolinostat treated	B2M	SYBR green	18,93	81,50	2641,25	77,00	93,00
B13	K562	Cell line	10 µM Ricolinostat treated	B2M	SYBR green	18,41	81,50	2482,18	77,00	93,00
B14	K562	Cell line	10 µM Ricolinostat treated	B2M	SYBR green	18,36	81,50	2558,68	77,00	95,00
B15	K562	Cell line	10 µM Ricolinostat treated	B2M	SYBR green	18,40	81,50	2623,72	77,50	95,00
C01	K562	Cell line	DMSO treated	Beta-Actin	SYBR green	15,05	84,50	2670,24	77,50	92,50
C02	K562	Cell line	DMSO treated	Beta-Actin	SYBR green	15,11	84,50	2708,63	77,50	93,50
C03	K562	Cell line	DMSO treated	Beta-Actin	SYBR green	15,20	84,50	2743,12	77,50	93,00
C10	K562	Cell line	5 µM Ricolinostat treated	Beta-Actin	SYBR green	15,35	84,50	2833,45	78,00	94,00
C11	K562	Cell line	5 µM Ricolinostat treated	Beta-Actin	SYBR green	15,40	84,50	2783,44	78,00	94,00
C12	K562	Cell line	5 µM Ricolinostat treated	Beta-Actin	SYBR green	15,52	84,50	2878,44	78,00	94,00
C13	K562	Cell line	10 µM Ricolinostat treated	Beta-Actin	SYBR green	15,31	84,50	2812,18	78,00	95,00
C14	K562	Cell line	10 µM Ricolinostat treated	Beta-Actin	SYBR green	15,27	84,50	2073,85	78,00	94,00
C15	K562	Cell line	10 µM Ricolinostat treated	Beta-Actin	SYBR green	15,74	84,50	2945,23	77,50	93,00

Well	Sample	Sample type	Condition	Target	Dye	Cq	Melt Temperature	Peak Height	Begin Temperature	End Temperature
A01	PDX AML7	PDX	DMSO treated	RNase T2	SYBR green	23,05	85,00	1850,40	80,50	91,50
A02	PDX AML7	PDX	DMSO treated	RNase T2	SYBR green	22,66	85,00	1870,47	80,50	93,50
A03	PDX AML7	PDX	DMSO treated	RNase T2	SYBR green	22,72	85,00	1853,58	81,00	92,50
A04	PDX AML7	PDX	0.25 µM Ricolinostat treated	RNase T2	SYBR green	23,68	85,00	1709,38	81,00	93,00
A05	PDX AML7	PDX	0.25 µM Ricolinostat treated	RNase T2	SYBR green	23,20	85,50	1632,97	81,00	91,00
A06	PDX AML7	PDX	0.25 µM Ricolinostat treated	RNase T2	SYBR green	23,43	85,50	1661,93	81,00	93,00
A07	PDX AML7	PDX	0.5 µM Ricolinostat treated	RNase T2	SYBR green	24,26	85,00	1519,32	81,00	92,50
A08	PDX AML7	PDX	0.5 µM Ricolinostat treated	RNase T2	SYBR green	23,77	85,50	1482,66	81,50	93,50
A09	PDX AML7	PDX	0.5 µM Ricolinostat treated	RNase T2	SYBR green	24,16	85,00	1437,91	81,00	92,00
A10	PDX AML7	PDX	1 µM Ricolinostat treated	RNase T2	SYBR green	24,63	85,50	1550,18	81,50	92,00
A11	PDX AML7	PDX	1 µM Ricolinostat treated	RNase T2	SYBR green	24,57	85,50	1437,95	81,50	94,00
A12	PDX AML7	PDX	1 µM Ricolinostat treated	RNase T2	SYBR green	24,97	85,00	1373,43	81,50	92,50
A13	PDX AML7	PDX	0.25 µM Citarinostat treated	RNase T2	SYBR green	22,62	85,50	1882,67	81,00	95,00
A14	PDX AML7	PDX	0.25 µM Citarinostat treated	RNase T2	SYBR green	22,66	85,50	1779,36	81,00	94,00
A15	PDX AML7	PDX	0.25 µM Citarinostat treated	RNase T2	SYBR green	22,74	85,50	1798,08	81,00	93,00
A16	PDX AML7	PDX	0.5 µM Citarinostat treated	RNase T2	SYBR green	23,88	85,00	1498,33	81,00	93,00
A17	PDX AML7	PDX	0.5 µM Citarinostat treated	RNase T2	SYBR green	23,55	85,50	1649,96	81,00	92,00
A18	PDX AML7	PDX	0.5 µM Citarinostat treated	RNase T2	SYBR green	23,52	85,00	1656,72	81,00	93,50
A19	PDX AML7	PDX	1 µM Citarinostat treated	RNase T2	SYBR green	24,87	85,50	1445,32	81,50	95,00
A20	PDX AML7	PDX	1 µM Citarinostat treated	RNase T2	SYBR green	24,80	85,50	1604,65	81,50	92,00
A21	PDX AML7	PDX	1 µM Citarinostat treated	RNase T2	SYBR green	24,66	85,00	1374,13	81,00	91,00
D01	PDX AML7	PDX	DMSO treated	B2M	SYBR green	21,64	81,50	2260,19	77,00	95,00
D02	PDX AML7	PDX	DMSO treated	B2M	SYBR green	21,55	81,50	2458,68	77,00	93,50
D03	PDX AML7	PDX	DMSO treated	B2M	SYBR green	21,55	81,50	2457,68	77,50	93,00
D04	PDX AML7	PDX	0.25 µM Ricolinostat treated	B2M	SYBR green	23,31	81,50	2174,71	77,50	85,50
D05	PDX AML7	PDX	0.25 µM Ricolinostat treated	B2M	SYBR green	23,19	81,50	2455,69	77,50	93,00
D06	PDX AML7	PDX	0.25 µM Ricolinostat treated	B2M	SYBR green	23,42	81,50	2435,88	77,50	94,00
D07	PDX AML7	PDX	0.5 µM Ricolinostat treated	B2M	SYBR green	24,50	81,50	2382,13	77,50	85,50
D08	PDX AML7	PDX	0.5 µM Ricolinostat treated	B2M	SYBR green	24,65	81,50	2141,59	77,50	94,00
D09	PDX AML7	PDX	0.5 µM Ricolinostat treated	B2M	SYBR green	24,77	81,50	2083,90	77,50	92,00
D10	PDX AML7	PDX	1 µM Ricolinostat treated	B2M	SYBR green	25,65	81,50	2335,67	77,50	91,50
D11	PDX AML7	PDX	1 µM Ricolinostat treated	B2M	SYBR green	26,02	81,50	2325,55	77,50	93,50
D12	PDX AML7	PDX	1 µM Ricolinostat treated	B2M	SYBR green	26,20	81,50	2385,80	77,50	92,00
D13	PDX AML7	PDX	0.25 µM Citarinostat treated	B2M	SYBR green	22,04	81,50	2221,50	77,50	92,50
D14	PDX AML7	PDX	0.25 µM Citarinostat treated	B2M	SYBR green	21,98	81,50	2415,27	77,50	95,00
D15	PDX AML7	PDX	0.25 µM Citarinostat treated	B2M	SYBR green	21,83	81,50	2317,60	77,50	95,00
D16	PDX AML7	PDX	0.5 µM Citarinostat treated	B2M	SYBR green	24,43	81,50	2088,31	77,50	94,00
D17	PDX AML7	PDX	0.5 µM Citarinostat treated	B2M	SYBR green	24,78	81,50	2171,95	77,00	92,00
D18	PDX AML7	PDX	0.5 µM Citarinostat treated	B2M	SYBR green	24,58	81,50	2231,28	77,00	85,50
D19	PDX AML7	PDX	1 µM Citarinostat treated	B2M	SYBR green	26,27	81,50	2145,58	77,50	92,50
D20	PDX AML7	PDX	1 µM Citarinostat treated	B2M	SYBR green	27,12	81,50	2034,58	77,50	92,00
D21	PDX AML7	PDX	1 µM Citarinostat treated	B2M	SYBR green	26,12	81,50	2151,45	77,50	85,50

E01	PDX AML7	PDX	DMSO treated	GAPDH	SYBR green	19,79	84,50	1559,61	80,50	95,00
E02	PDX AML7	PDX	DMSO treated	GAPDH	SYBR green	19,59	84,50	1729,02	80,50	91,00
E03	PDX AML7	PDX	DMSO treated	GAPDH	SYBR green	19,63	84,50	1915,23	80,50	93,00
E04	PDX AML7	PDX	0.25 µM Ricolinostat treated	GAPDH	SYBR green	19,64	84,50	1831,48	80,50	94,00
E05	PDX AML7	PDX	0.25 µM Ricolinostat treated	GAPDH	SYBR green	20,40	84,50	1777,23	80,50	90,50
E06	PDX AML7	PDX	0.25 µM Ricolinostat treated	GAPDH	SYBR green	19,82	84,50	1815,13	80,50	91,50
E07	PDX AML7	PDX	0.5 µM Ricolinostat treated	GAPDH	SYBR green	20,16	84,50	1776,67	80,50	91,50
E08	PDX AML7	PDX	0.5 µM Ricolinostat treated	GAPDH	SYBR green	20,76	84,50	1740,72	80,50	92,00
E09	PDX AML7	PDX	0.5 µM Ricolinostat treated	GAPDH	SYBR green	20,22	84,50	1772,67	80,50	91,00
E10	PDX AML7	PDX	1 µM Ricolinostat treated	GAPDH	SYBR green	20,33	84,50	1805,66	80,50	92,00
E11	PDX AML7	PDX	1 µM Ricolinostat treated	GAPDH	SYBR green	21,26	84,50	1805,15	80,50	94,00
E12	PDX AML7	PDX	1 µM Ricolinostat treated	GAPDH	SYBR green	20,70	85,00	1830,90	80,50	94,00
E13	PDX AML7	PDX	0.25 µM Citarinostat treated	GAPDH	SYBR green	19,45	84,50	1894,41	80,50	94,00
E14	PDX AML7	PDX	0.25 µM Citarinostat treated	GAPDH	SYBR green	19,95	84,50	1843,89	80,50	95,00
E15	PDX AML7	PDX	0.25 µM Citarinostat treated	GAPDH	SYBR green	19,23	84,50	1739,44	80,50	93,00
E16	PDX AML7	PDX	0.5 µM Citarinostat treated	GAPDH	SYBR green	20,13	84,50	1885,70	80,50	94,00
E17	PDX AML7	PDX	0.5 µM Citarinostat treated	GAPDH	SYBR green	20,10	84,50	1876,47	80,50	94,00
E18	PDX AML7	PDX	0.5 µM Citarinostat treated	GAPDH	SYBR green	20,33	84,50	1791,33	80,50	93,00
E19	PDX AML7	PDX	1 µM Citarinostat treated	GAPDH	SYBR green	21,14	84,50	1616,56	80,50	92,00
E20	PDX AML7	PDX	1 µM Citarinostat treated	GAPDH	SYBR green	21,66	84,50	1663,83	80,50	90,50
E21	PDX AML7	PDX	1 µM Citarinostat treated	GAPDH	SYBR green	20,99	84,50	1549,52	80,50	90,50

Well	Sample	Sample type	Condition	Target	Dye	Cq	Melt Temperature	Peak Height	Begin Temperature	End Temperature
A22	THP1	Cell line	DMSO treated	RNase T2	SYBR green	20,15	85,00	2060,34	80,50	95,00
A23	THP1	Cell line	DMSO treated	RNase T2	SYBR green	20,21	85,00	1704,47	80,50	95,00
A24	THP1	Cell line	DMSO treated	RNase T2	SYBR green	20,33	85,00	1741,66	80,50	91,50
F01	THP1	Cell line	0.25 µM Ricolinostat treated	RNase T2	SYBR green	21,99	85,50	2003,74	80,00	91,00
F02	THP1	Cell line	0.25 µM Ricolinostat treated	RNase T2	SYBR green	21,22	85,50	2300,79	80,00	95,00
F03	THP1	Cell line	0.25 µM Ricolinostat treated	RNase T2	SYBR green	22,04	85,50	2160,15	80,50	93,50
F04	THP1	Cell line	0.5 µM Ricolinostat treated	RNase T2	SYBR green	19,99	85,50	2218,69	80,50	94,00
F05	THP1	Cell line	0.5 µM Ricolinostat treated	RNase T2	SYBR green	19,74	85,50	2308,57	80,50	95,00
F06	THP1	Cell line	0.5 µM Ricolinostat treated	RNase T2	SYBR green	20,43	85,50	2058,23	80,50	92,50
F07	THP1	Cell line	1 µM Ricolinostat treated	RNase T2	SYBR green	20,16	85,50	2220,51	80,50	94,00
F08	THP1	Cell line	1 µM Ricolinostat treated	RNase T2	SYBR green	20,05	85,50	2264,37	80,50	93,00
F09	THP1	Cell line	1 µM Ricolinostat treated	RNase T2	SYBR green	20,30	85,50	2309,93	80,50	94,00
F10	THP1	Cell line	0.25 µM Citarinostat treated	RNase T2	SYBR green	19,81	85,50	2294,53	80,50	94,00
F11	THP1	Cell line	0.25 µM Citarinostat treated	RNase T2	SYBR green	19,97	85,50	2284,43	80,50	92,00
F12	THP1	Cell line	0.25 µM Citarinostat treated	RNase T2	SYBR green	20,12	85,50	2132,68	80,50	93,00
F13	THP1	Cell line	0.5 µM Citarinostat treated	RNase T2	SYBR green	19,66	85,50	2287,18	80,50	94,00
F14	THP1	Cell line	0.5 µM Citarinostat treated	RNase T2	SYBR green	19,84	85,50	2316,37	80,50	93,50
F15	THP1	Cell line	0.5 µM Citarinostat treated	RNase T2	SYBR green	20,11	85,50	2334,18	80,50	93,50
F16	THP1	Cell line	1 µM Citarinostat treated	RNase T2	SYBR green	20,18	85,50	2165,17	80,50	94,00
F17	THP1	Cell line	1 µM Citarinostat treated	RNase T2	SYBR green	20,23	85,50	2289,82	80,50	93,00
F18	THP1	Cell line	1 µM Citarinostat treated	RNase T2	SYBR green	20,75	85,50	2022,05	80,50	94,00
D22	THP1	Cell line	DMSO treated	B2M	SYBR green	18,24	81,50	2193,40	77,50	92,00
D23	THP1	Cell line	DMSO treated	B2M	SYBR green	18,19	81,50	2179,97	77,00	95,00
D24	THP1	Cell line	DMSO treated	B2M	SYBR green	18,49	81,50	2335,57	77,50	95,00
I01	THP1	Cell line	0.25 µM Ricolinostat treated	B2M	SYBR green	20,44	81,50	2306,43	77,50	88,50
I02	THP1	Cell line	0.25 µM Ricolinostat treated	B2M	SYBR green	20,10	81,50	2577,09	77,00	91,00
I03	THP1	Cell line	0.25 µM Ricolinostat treated	B2M	SYBR green	20,51	81,50	2578,65	77,50	92,00
I04	THP1	Cell line	0.5 µM Ricolinostat treated	B2M	SYBR green	18,21	81,50	2678,00	77,00	95,00
I05	THP1	Cell line	0.5 µM Ricolinostat treated	B2M	SYBR green	18,27	81,50	2671,80	77,50	94,00
I06	THP1	Cell line	0.5 µM Ricolinostat treated	B2M	SYBR green	18,79	81,50	2493,85	77,00	94,00
I07	THP1	Cell line	1 µM Ricolinostat treated	B2M	SYBR green	18,39	81,50	2607,44	77,50	94,00
I08	THP1	Cell line	1 µM Ricolinostat treated	B2M	SYBR green	18,39	81,50	2555,15	77,00	93,50
I09	THP1	Cell line	1 µM Ricolinostat treated	B2M	SYBR green	18,56	81,50	2530,84	77,50	93,50
I10	THP1	Cell line	0.25 µM Citarinostat treated	B2M	SYBR green	18,37	81,50	2689,60	77,00	95,00
I11	THP1	Cell line	0.25 µM Citarinostat treated	B2M	SYBR green	18,41	81,50	2422,75	77,00	92,50
I12	THP1	Cell line	0.25 µM Citarinostat treated	B2M	SYBR green	18,29	81,50	2525,75	77,00	94,00
I13	THP1	Cell line	0.5 µM Citarinostat treated	B2M	SYBR green	18,35	81,50	2521,88	77,50	92,50
I14	THP1	Cell line	0.5 µM Citarinostat treated	B2M	SYBR green	18,52	81,50	2589,65	77,00	93,00
I15	THP1	Cell line	0.5 µM Citarinostat treated	B2M	SYBR green	18,59	81,50	2744,80	77,00	93,00
I16	THP1	Cell line	1 µM Citarinostat treated	B2M	SYBR green	18,46	81,50	2644,22	77,00	94,00
I17	THP1	Cell line	1 µM Citarinostat treated	B2M	SYBR green	19,15	81,50	2199,33	77,00	92,00
I18	THP1	Cell line	1 µM Citarinostat treated	B2M	SYBR green	18,82	81,50	2476,33	77,00	92,00

E22	THP1	Cell line	DMSO treated	GAPDH	SYBR green	18,21	84,50	1493,61	80,50	93,50
E23	THP1	Cell line	DMSO treated	GAPDH	SYBR green	18,51	84,50	1593,26	80,50	93,50
E24	THP1	Cell line	DMSO treated	GAPDH	SYBR green	18,19	84,50	1533,94	80,50	93,50
J01	THP1	Cell line	0.25 µM Ricolinostat treated	GAPDH	SYBR green	20,32	84,50	1482,97	80,50	91,00
J02	THP1	Cell line	0.25 µM Ricolinostat treated	GAPDH	SYBR green	20,05	84,50	1716,13	80,50	94,00
J03	THP1	Cell line	0.25 µM Ricolinostat treated	GAPDH	SYBR green	20,07	84,50	1732,61	80,50	92,50
J04	THP1	Cell line	0.5 µM Ricolinostat treated	GAPDH	SYBR green	18,45	84,50	1709,67	80,50	93,50
J05	THP1	Cell line	0.5 µM Ricolinostat treated	GAPDH	SYBR green	19,16	84,50	1634,31	80,50	93,00
J06	THP1	Cell line	0.5 µM Ricolinostat treated	GAPDH	SYBR green	18,78	84,50	1715,23	80,50	93,00
J07	THP1	Cell line	1 µM Ricolinostat treated	GAPDH	SYBR green	18,89	84,50	1677,85	80,50	93,00
J08	THP1	Cell line	1 µM Ricolinostat treated	GAPDH	SYBR green	19,59	84,50	1736,47	80,50	92,00
J09	THP1	Cell line	1 µM Ricolinostat treated	GAPDH	SYBR green	19,03	84,50	1763,35	80,50	94,00
J10	THP1	Cell line	0.25 µM Citarinostat treated	GAPDH	SYBR green	18,33	84,50	2024,85	80,50	93,00
J11	THP1	Cell line	0.25 µM Citarinostat treated	GAPDH	SYBR green	19,01	84,50	1671,75	80,50	91,00
J12	THP1	Cell line	0.25 µM Citarinostat treated	GAPDH	SYBR green	18,36	84,50	1710,04	80,50	93,00
J13	THP1	Cell line	0.5 µM Citarinostat treated	GAPDH	SYBR green	18,53	84,50	1650,57	80,50	94,00
J14	THP1	Cell line	0.5 µM Citarinostat treated	GAPDH	SYBR green	18,71	84,50	1779,25	80,50	92,00
J15	THP1	Cell line	0.5 µM Citarinostat treated	GAPDH	SYBR green	18,57	84,50	1755,65	80,50	94,00
J16	THP1	Cell line	1 µM Citarinostat treated	GAPDH	SYBR green	19,13	84,50	1747,54	80,50	93,50
J17	THP1	Cell line	1 µM Citarinostat treated	GAPDH	SYBR green	19,51	84,50	1663,69	80,50	92,00
J18	THP1	Cell line	1 µM Citarinostat treated	GAPDH	SYBR green	19,32	84,50	1690,96	80,50	92,00

Well	Sample	Sample type	Condition	Target	Dye	Cq	Melt Temperature	Peak Height	Begin Temperature	End Temperature
F19	PDX CML	PDX	DMSO treated	RNase T2	SYBR green	30,12	85,50	1679,27	80,50	92,50
F20	PDX CML	PDX	DMSO treated	RNase T2	SYBR green	29,49	85,50	1730,67	80,50	92,50
F21	PDX CML	PDX	DMSO treated	RNase T2	SYBR green	29,30	85,50	1699,98	80,50	91,50
F22	PDX CML	PDX	0.25 µM Ricolinostat treated	RNase T2	SYBR green	30,64	85,50	1462,57	81,00	91,50
F23	PDX CML	PDX	0.25 µM Ricolinostat treated	RNase T2	SYBR green	30,38	85,50	1765,10	81,00	91,50
F24	PDX CML	PDX	0.25 µM Ricolinostat treated	RNase T2	SYBR green	30,70	85,50	1302,45	81,00	93,50
K01	PDX CML	PDX	0.5 µM Ricolinostat treated	RNase T2	SYBR green	31,02	85,50	1651,11	80,50	91,50
K02	PDX CML	PDX	0.5 µM Ricolinostat treated	RNase T2	SYBR green	30,41	85,50	1764,71	79,50	95,00
K03	PDX CML	PDX	0.5 µM Ricolinostat treated	RNase T2	SYBR green	30,60	85,50	1670,08	81,50	91,00
K04	PDX CML	PDX	1 µM Ricolinostat treated	RNase T2	SYBR green	33,19	85,50	1675,06	81,50	91,00
K05	PDX CML	PDX	1 µM Ricolinostat treated	RNase T2	SYBR green	32,42	85,50	1401,59	81,50	90,50
K06	PDX CML	PDX	1 µM Ricolinostat treated	RNase T2	SYBR green	32,64	85,50	1364,33	81,00	91,00
I19	PDX CML	PDX	DMSO treated	B2M	SYBR green	25,59	81,50	2116,21	77,50	85,50
I20	PDX CML	PDX	DMSO treated	B2M	SYBR green	25,62	81,50	2475,06	77,50	87,00
I21	PDX CML	PDX	DMSO treated	B2M	SYBR green	25,84	81,50	2193,81	77,50	88,00
I22	PDX CML	PDX	0.25 µM Ricolinostat treated	B2M	SYBR green	27,00	81,50	2123,33	77,50	85,50
I23	PDX CML	PDX	0.25 µM Ricolinostat treated	B2M	SYBR green	27,36	81,50	2058,20	77,50	85,50
I24	PDX CML	PDX	0.25 µM Ricolinostat treated	B2M	SYBR green	27,44	81,50	2022,58	77,50	88,50
N01	PDX CML	PDX	0.5 µM Ricolinostat treated	B2M	SYBR green	28,47	81,50	2049,93	77,50	85,50
N02	PDX CML	PDX	0.5 µM Ricolinostat treated	B2M	SYBR green	28,62	81,50	2042,80	77,50	86,00
N03	PDX CML	PDX	0.5 µM Ricolinostat treated	B2M	SYBR green	28,72	81,50	1834,13	77,50	86,50
N04	PDX CML	PDX	1 µM Ricolinostat treated	B2M	SYBR green	33,31	81,50	1557,37	78,00	85,50
N05	PDX CML	PDX	1 µM Ricolinostat treated	B2M	SYBR green	32,85	81,50	1659,12	78,00	85,50
N06	PDX CML	PDX	1 µM Ricolinostat treated	B2M	SYBR green	33,61	82,00	1573,65	78,00	85,50
J19	PDX CML	PDX	DMSO treated	GAPDH	SYBR green	26,27	84,50	1354,41	79,50	91,50
J20	PDX CML	PDX	DMSO treated	GAPDH	SYBR green	26,39	84,50	1317,75	79,50	89,00
J21	PDX CML	PDX	DMSO treated	GAPDH	SYBR green	26,58	84,50	1322,31	79,50	92,00
J22	PDX CML	PDX	0.25 µM Ricolinostat treated	GAPDH	SYBR green	28,05	84,50	1322,49	79,50	92,00
J23	PDX CML	PDX	0.25 µM Ricolinostat treated	GAPDH	SYBR green	28,19	84,50	1329,94	79,50	92,00
J24	PDX CML	PDX	0.25 µM Ricolinostat treated	GAPDH	SYBR green	28,36	84,50	1356,99	79,50	92,50
O01	PDX CML	PDX	0.5 µM Ricolinostat treated	GAPDH	SYBR green	29,88	84,50	1345,85	79,50	92,00
O02	PDX CML	PDX	0.5 µM Ricolinostat treated	GAPDH	SYBR green	29,36	84,50	1345,20	79,50	92,50
O03	PDX CML	PDX	0.5 µM Ricolinostat treated	GAPDH	SYBR green	28,95	84,50	1302,21	79,50	92,50
O04	PDX CML	PDX	1 µM Ricolinostat treated	GAPDH	SYBR green	31,15	84,50	1313,97	79,50	92,50
O05	PDX CML	PDX	1 µM Ricolinostat treated	GAPDH	SYBR green	32,42	84,50	1323,23	79,50	92,50
O06	PDX CML	PDX	1 µM Ricolinostat treated	GAPDH	SYBR green	31,78	84,50	1345,18	79,50	92,50
